# Exogenous Epstein–Barr virus nuclear antigen 1 induces ADAR1-driven tumor resistance against immunotherapy

**DOI:** 10.1038/s41392-026-02574-y

**Published:** 2026-02-18

**Authors:** Changlin Liu, Zhiqiang Sun, Chao Li, Yanqing Zhou, Xuefeng Gao, Yuping Zhong, Xiaomin Luo, Chenci Wang, Yuanbin Zhang, Chuping Ni, Manli Peng, Weiquan Jian, Yinggui Yang, Xuewen Zhang, Yichang Ren, Xinqi Gong, Min Zhao, Xia Guo, Chao Cheng, Jianjun Chen, Xin Li

**Affiliations:** 1 Shenzhen Key Laboratory of Viral Oncology, Shenzhen Hospital of Southern Medical University, Guangdong, China; 2 Department of Hematology, Nanfang Hospital, Southern Medical University, Guangzhou, Guangdong, China; 3 School of Pharmaceutical Science, Southern Medical University, Guangdong, China; 4 The Seventh Affiliated Hospital, Southern Medical University, Foshan, Guangdong P. R. China; 5 Department of Oncology, The Fifth Affiliated Hospital, Southern Medical University, Guangzhou, Guangdong China; 6 The Third School of Clinical Medicine, Southern Medical University, Guangzhou, Guangdong, China; 7 Department of Otolaryngology, Shenzhen Longgang Otolaryngology Hospital, Shenzhen, Guangdong China; 8 Department of Oncology, Funan County People’s Hospital, Fuyang, Anhui China; 9 Mathematical Intelligence Application LAB, Institute for Mathematical Sciences, Renmin University of China, Beijing China; 10 PANACRO (Hefei) Pharmaceutical Technology Co. Ltd., Hefei, China

**Keywords:** Head and neck cancer, Drug development, Immunotherapy, Tumour immunology, Tumour virus infections

## Abstract

Immune checkpoint blockade (ICB) therapy continues to face limitations due to tumor resistance linked to suppressed interferon (IFN) signaling. This suppression can be attributed to multiple mechanisms, among which viral pathogens represent a compelling though not yet fully elucidated factor. Here, we demonstrate that exogenous Epstein–Barr virus-encoded EBNA1 drives immunosuppression via enhanced RNA-editing enzyme ADAR1-mediated RNA editing. Comparative tumor model analyses revealed that EBNA1 overexpression reduced CD8^+^ T-cell infiltration, inhibited IFN responses, polarized macrophages toward the M2 phenotype, and accelerated tumor growth. Mechanistically, EBNA1 forms a trimeric complex with insulin-like growth factor 2 mRNA-binding protein 3 (IGF2BP3) and eukaryotic translation initiation factor 4G1 (EIF4G1), enhancing ADAR1 translation. Elevated ADAR1 further increased A-to-I editing of dsRNA, particularly within SINE elements near IFN-associated genes. This editing masked immunostimulatory signals, impairing RNA sensor activation and blunting IFN pathways. Notably, combining the EBNA1-targeting PROTAC degrader EP-1215 with anti-PD-1 effectively restored IFN signaling, enhanced T-cell infiltration, and suppressed EBNA1^+^ tumors in humanized mice. This viral exploitation of RNA editing suggests that targeting EBNA1 could be a strategy to convert “cold” tumors into “hot” targets amenable to ICB therapy.

## Introduction

Immune checkpoint blockade (ICB) therapy has revolutionized the treatment landscape of multiple malignancies and has demonstrated remarkable clinical efficacy in a subset of patients. However, despite its transformative potential, only approximately 20–30% of cancer patients derive durable clinical benefit from ICB therapy, highlighting a major unmet challenge in tumor immunotherapy.^1–3^ As immunotherapeutic strategies are increasingly incorporated into standard-of-care regimens across cancer types, both primary and acquired resistance to immune checkpoint inhibitors have become more evident. Consequently, understanding the molecular and immunological mechanisms underlying ICB resistance remains a critical priority. To address this limitation, extensive efforts have been devoted to the development of combination therapeutic strategies, integrating immune checkpoint inhibitors with chemotherapy, radiotherapy, targeted therapy, or novel immunomodulatory agents.^4–7^ These approaches are particularly important for patients with recurrent or metastatic disease who exhibit resistance to PD-1 or PD-L1 blockade. A key factor contributing to resistance to ICB therapy is the low infiltration of immune cells, especially CD8^+^ T cells and natural killer cells, in “cold” tumors.^8^ Conversely, “hot” tumors are characterized by high levels of T-cell infiltration and are generally more responsive to ICB. Therefore, identifying effective combination strategies with immune checkpoint inhibitors to convert “cold” tumors to “hot” tumors is crucial for overcoming immunotherapy resistance.

RNA editing has recently emerged as an important regulatory layer in tumor immunity. Among RNA-modifying enzymes, adenosine deaminase acting on RNA 1 (ADAR1) has gained considerable attention due to its pivotal role in modulating innate immune sensing. ADAR1 catalyzes the conversion of adenosine to inosine (A-to-I) within double-stranded RNA (dsRNA), thereby preventing aberrant activation of antiviral immune pathways. Several influential studies have demonstrated that loss of ADAR1 sensitizes tumors to immune-mediated destruction and enhances responsiveness to immune checkpoint blockade.^9–11^ Mechanistically, in the absence of ADAR1-mediated RNA editing, unedited endogenous dsRNAs accumulate in the cytoplasm and are recognized by pattern recognition receptors such as MDA5 and PKR. This recognition triggers robust activation of type I interferons (IFNs) signaling, leading to induction of interferon-stimulated genes (ISGs), amplification of inflammatory responses, and enhanced antitumor immunity. Thus, ADAR1 functions as a critical immune checkpoint at the level of innate immune sensing, enabling tumor cells to evade immune surveillance by neutralizing the immunogenicity of endogenous dsRNAs. Nevertheless, while the tumor-intrinsic role of ADAR1 in immune resistance has been increasingly appreciated, the impact of exogenous viral factors on ADAR1-mediated immune regulation remains poorly understood.

Epstein–Barr virus (EBV) is a ubiquitous oncogenic herpesvirus etiologically linked to several human malignancies, including nasopharyngeal carcinoma (NPC), gastric carcinoma, and multiple subtypes of lymphoma.^12,13^ NPC is a prototypical EBV-associated epithelial cancer and represents an ideal model for investigating virus-driven immune evasion. Among EBV-encoded latent proteins, Epstein–Barr virus nuclear antigen 1 (EBNA1) is uniquely and consistently expressed in all EBV-positive NPC cells.^14^ It plays a crucial role in the latent phase of EBV infection, aiding in viral replication and ensuring stable viral persistence within host cells.^15^ It also activates other essential EBV latent genes involved in cellular immortalization.^16^ Through interactions with key cellular proteins such as USP7 and the MCM complex,^17,18^ EBNA1 manages multiple pathways important for viral persistence and cell survival, contributing to tumor development and spread. In addition to these functions, earlier studies suggest that EBNA1 may facilitate immune evasion in NPC. Although EBV latent proteins typically elicit strong immune responses, cells expressing EBNA1 alone can largely evade immune surveillance.^19^ This effect may be partly due to EBNA1-mediated recruitment of Treg cells^20,21^ and its glycine–alanine repeat (Gar) domain inhibiting the MHC class I presentation of EBNA1 epitopes to cytotoxic T cells.^22^ Nevertheless, the mechanisms through which EBNA1 modulates the tumor immune microenvironment, particularly those involving ADAR1-mediated resistance to immunotherapy, remain incompletely understood and merit further investigation.

In this study, we demonstrated that exogenous EBNA1 expression induces an immunosuppressive tumor microenvironment in nasopharyngeal carcinoma by functionally engaging the ADAR1 pathway, thereby promoting resistance to immunotherapy. By systematically dissecting EBNA1-associated protein–RNA interactions, we revealed how EBNA1 hijacks host RNA-editing machinery within the established framework of ADAR1-mediated suppression of dsRNA sensing. Importantly, we developed a proteolysis-targeting chimera (PROTAC) degrader that specifically targets EBNA1 and effectively disrupts EBNA1-driven immune suppression. When combined with PD-1 antibody therapy, EBNA1 degradation restores interferon signaling, enhances immune cell infiltration, and significantly improves antitumor efficacy in EBV-positive NPC models. Collectively, our findings identify the EBNA1–ADAR1 axis as a central mediator of immunosuppression and propose a novel PROTAC-based therapeutic strategy to overcome immune checkpoint blockade resistance in EBV-associated malignancies.

## Results

### Exogenous EBNA1 induces a tumor immunosuppressive microenvironment

**Fig. 1:**
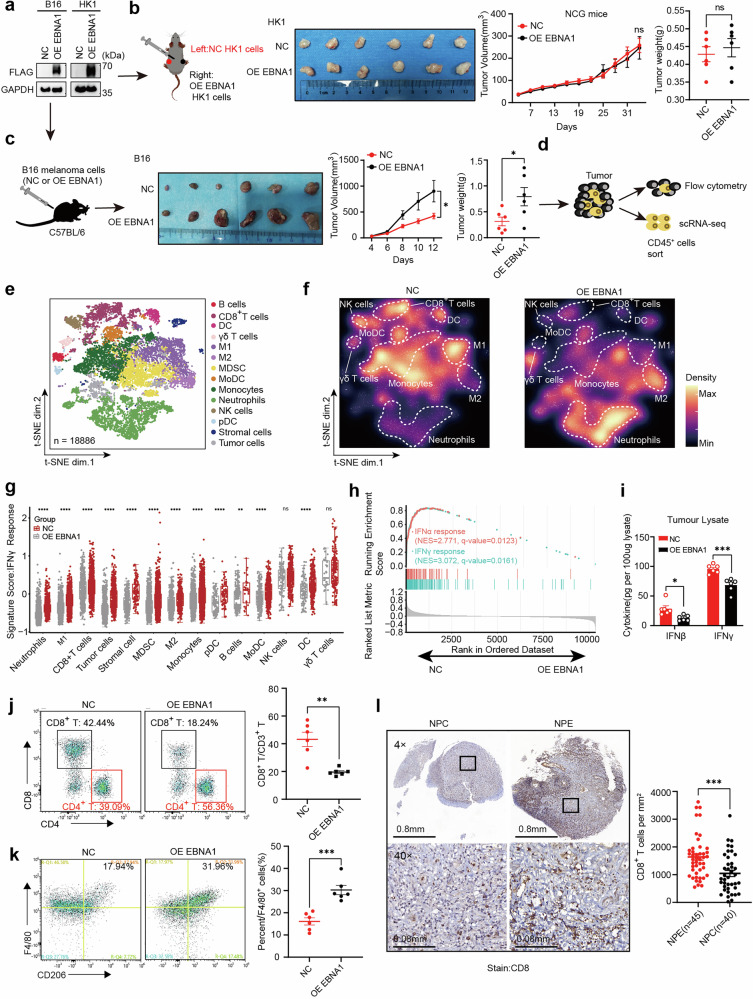
EBNA1 modulates the tumor microenvironment by suppressing inflammatory responses. **a** EBNA1 protein was overexpressed in both melanoma B16 and NPC HK1 cells. NC cells represent control tumor cells, and OE EBNA1 cells represent EBNA1-overexpressing tumor cells. The data are representative of three independent experiments. **b** Representative images of subcutaneous tumors in severe immunodeficient NCG mice, including HK1-NC tumors (left) and HK1-OE EBNA1 tumors (right), with corresponding tumor volumes and weights. The mouse diagram was created via FigDraw. The data are presented as the means ± s.e.m.s (n = 6 mice in each group). **c** Representative images of tumors from C57BL/6 mice injected with either NC or OE EBNA1 B16 cells (left), the corresponding tumor volume (middle), and the tumor weight (right) are shown. The mouse diagram was created via FigDraw. The data are shown as the means ± s.e.m.s n = 6 mice per group. **d** Experimental design for single-cell analysis or FACS validation of CD45^+^ cells sorted from tumors with or without EBNA1 expression. **e**, **f** t-distributed stochastic neighbor embedding (t-SNE) plot (**c**) and density plot (**d**) of 18,886 RNA sequencing (RNA-seq) single CD45^+^ cells from B16-OE EBNA1 and B16-NC tumors. M1, M1 macrophages; M2, M2 macrophages; cDCs, conventional dendritic cells; MoDCs, monocyte-derived dendritic cells; pDCs, plasmacytoid dendritic cells. **g** Single-cell gene set enrichment scores of the IFNγ response signature in individual immune subsets from EBNA1-overexpressing and control tumors (P-values were calculated via the Kolmogorov‒Smirnov test). n = 18,886 cells. **h** Gene set enrichment analysis (GSEA) was performed on immune cells from EBNA1-overexpressing tumors and control tumors to analyze the interferon response signature. **i** Protein levels of IFNβ and IFNγ in the TME of EBNA1 overexpressing and control B16 tumors. n = 6 in each group. **j** Flow cytometry analysis of the proportion of CD8^+^ T cells in the tumor microenvironment using the tumor-derived cells shown in (**d**). Each group consisted of 6 samples (n = 6). **k** Flow cytometry analysis of the proportion of M2 macrophages in the tumor microenvironment using the tumor-derived cells shown in (**d**). There were 6 samples per group (n = 6). **l** Immunohistochemistry (left) and quantification (right) of CD8^+^ T cells in NPC patient samples (black, n = 40) or NPE samples (red, n = 45). **b** Growth curves, **c** Growth curves, two-way ANOVA. **b** Tumor weight. **c** Tumor weight. **i**‒**l** Two-tailed unpaired t-test; *P < 0.05; **P < 0.01; ****P < 0.001; ns, not significant

To understand the role of EBNA1 in tumor development, we overexpressed EBNA1 in HK1 NPC cells (Fig. 1a) and implanted them into immunocompromised NCG mice. EBNA1 did not increase tumor growth (Fig. 1b), which is consistent with previous studies suggesting that EBNA1 does not directly promote cellular proliferation or tumorigenesis,^23,24^ especially in the absence of an intact immune microenvironment. Conversely, when B16 murine melanoma cells overexpressing EBNA1 were introduced into immunocompetent C57BL/6 mice, they developed significantly larger tumors than controls did (Fig. 1c). Notably, this growth advantage was abolished in immunodeficient NCG mice (Supplementary Fig. 1a), indicating that the observed effect is immune-dependent. This finding was reproduced in the highly immunogenic CT26 colon carcinoma model, where EBNA1 similarly promoted tumor growth only in immunocompetent hosts (Supplementary Fig. 1b, c). In summary, EBNA1 may promote tumor progression through its interactions with the immune system rather than through intrinsic oncogenic activity.

To characterize this immune interaction, we performed single-cell RNA sequencing of CD45⁺ immune cells from B16 tumors (Fig. 1d). EBNA1-overexpressing tumors presented markedly reduced infiltration of immune-activating cells (CD8⁺ T cells, NK cells, neutrophils, and γδ T cells) and increased numbers of M2 macrophages (Fig. 1e, f; Supplementary Fig. 1d, e). Further analysis revealed that CD8⁺ T cells in EBNA1-overexpressing tumors exhibited an exhausted phenotype, with upregulated PDCD1 and LAG3 and downregulated IFNG and GZMB. Similarly, macrophages displayed pronounced M2 polarization, marked by elevated ARG1 and IL10 and decreased NOS2 and IL12 levels (Supplementary Fig. 1f). Compared with that in control tumors, the expression of immune suppressive phenotype genes in immune cells was consistently greater in EBNA1-overexpressing tumors, whereas the expression of immune activation phenotype genes in immune cells was lower (Supplementary Fig. 1g). Gene expression related to CD8^+^ T-cell activation and IFNγ responses was significantly diminished in EBNA1-overexpressing tumors (Supplementary Fig. 1g). This immunosuppressive signature was further supported by a broad attenuation in IFNγ and IFNα response pathways across nearly all immune cell types (Fig. 1g, h), as well as by reduced levels of IFNβ and IFNγ proteins in whole-tumor lysates (Fig. 1i). Given that tumor cells themselves can produce interferons,^9,25^ the observed reduction likely reflects impaired responses in both immune and tumor cells. Additionally, CD8^+^ T cells in EBNA1-overexpressing tumors presented G1-phase arrest (Supplementary Fig. 1h) and reduced infiltration compared with those in control tumors (Fig. 1j). Conversely, M2 macrophage infiltration was increased, whereas M1 macrophage infiltration remained unchanged (Fig. 1k, Supplementary Fig. 1i). Consistent with these experimental findings, analysis of clinical tissue microarrays revealed significantly decreased CD8⁺ T cells infiltration in NPC tissues (where EBNA1 was consistently detected) than in nasopharyngeal epithelial tissues (NPE), which presented no EBNA1 expression (Fig. 1l). This finding not only confirms that immune exclusion is a well-established characteristic of NPC,^26–28^ but also indirectly supports a potential association between EBNA1 and immunosuppression. Together, these data demonstrate that exogenous EBNA1 establishes a profoundly immunosuppressive tumor microenvironment, weakening antitumor immunity and potentially reducing the effectiveness of immunotherapy.

### EBNA1 interacts with the m^6^A reader IGF2BP3 to target ADAR1, which is potentially implicated in tumor immunosuppression

**Fig. 2:**
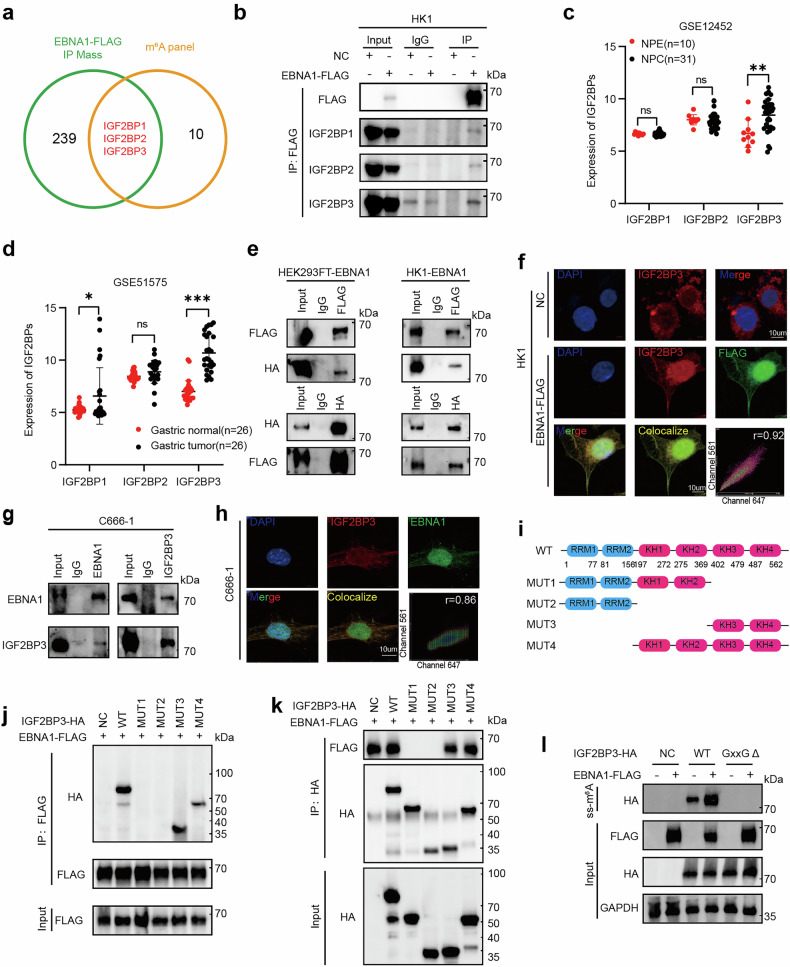
Interaction between EBNA1 and the m^6^A reader IGF2BP3. **a** Proteins that interact with EBNA1 were identified in HEK293FT cells through Co-IP and mass spectrometry, and Venn analysis was used to identify the proteins related to m^6^A modification. **b** The EBNA1-FLAG plasmid was transfected into HEK293FT cells, and the EBNA1-FLAG and cell lysate complexes were immunoprecipitated with an anti-FLAG antibody. The presence of IGF2BP1, IGF2BP2, and IGF2BP3 was then detected. NC: empty control plasmid, EBNA1-FLAG: EBNA1 overexpression plasmid. **c** Relative mRNA levels of IGF2BP1, IGF2BP2, and IGF2BP3 in normal (n = 10) and NPC (n = 31) tissue samples from the GSE12452 dataset. **d** Relative mRNA levels of IGF2BP1, IGF2BP2, and IGF2BP3 in normal (n = 26) and NPC (n = 26) tissue samples from the GSE51575 dataset. **e** The EBNA1-FLAG plasmid and IGF2BP3-HA plasmid were transfected into HEK293FT or HK1 cells. The EBNA1-FLAG and IGF2BP3-HA complexes were immunoprecipitated with anti-FLAG and anti-HA antibodies, and HA (IGF2BP3) and FLAG (EBNA1) were detected. The data are representative of two independent experiments. **f** Immunofluorescence assays using anti-FLAG and anti-IGF2BP3 antibodies were conducted to detect the colocalization of exogenous FLAG (representing EBNA1) and endogenous IGF2BP3 proteins in OE EBNA1 HK1 cells. Cellular nuclei were stained with DAPI and appeared blue. IGF2BP3 and FLAG are visualized in red and green, respectively. The merged image displays overlapping signals of DAPI, IGF2BP3, and FLAG, with colocalization indicated in yellow. Scatter analysis revealed signals in channels 647 (IGF2BP3) and 561 (FLAG). The Pearson correlation coefficient for colocalization is shown. Magnification: 100×. Scale bar = 10 µm. The data are representative of two independent experiments. **g** In C666-1 cells, the EBNA1–IGF2BP3 complex was immunoprecipitated via anti-EBNA1 and anti-IGF2BP3 antibodies, and IGF2BP3 and EBNA1 were detected. The data are representative of three independent experiments. **h** In C666-1 cells, immunofluorescence staining with anti-EBNA1 and anti-IGF2BP3 antibodies was performed to examine the colocalization of the EBNA1 and IGF2BP3 proteins. The cell nuclei were stained with DAPI and appeared blue. IGF2BP3 and EBNA1 are visualized in red and green, respectively. In the merged images, overlapping signals of DAPI, IGF2BP3, and EBNA1 appeared yellow, indicating colocalization. Scatter plot analysis revealed signals in channels 647 (IGF2BP3) and 561 (EBNA1). The Pearson correlation coefficient for colocalization is shown. Magnification: 100×. Scale bar = 10 µm. The data are representative of three independent experiments. **i** Diagram of the different domains in both the wild-type (WT) and mutant IGF2BP3 constructs. R represents the RRM. **j**, **k** The IGF2BP3-HA mutants and EBNA1-FLAG plasmids were cotransfected into HEK293FT cells. The EBNA1-FLAG (**h**) and IGF2BP3-HA (**i**) complexes were immunoprecipitated with anti-FLAG and anti-HA antibodies, respectively. The IGF2BP3-HA mutant and EBNA1-FLAG complexes were detected via anti-HA and anti-FLAG antibodies. The data are representative of two independent experiments. **l** Wild-type (WT) or GxxG mutant (GxxGΔ) IGF2BP3-HA plasmids were cotransfected with EBNA1-FLAG plasmids into HEK293FT cells. RNA pull-down assays were performed to study the in vitro binding of IGF2BP3 to single-stranded m^6^A-RNA probes. The data are representative of three independent experiments. **c**, **d** Data are shown as the mean ± s.e.m. Two-tailed unpaired t-tests were used for statistical analysis. *P < 0.05; **P < 0.01; ns, not significant

Given that NPC progression is driven primarily by epigenetic mechanisms,^29^ we focused on m^6^A modification-related proteins via Venn diagram analysis (Fig. 2a). This approach identified 250 potential interacting proteins, including 11 m^6^A-modifying factors. Among these, the IGF2BP family members (IGF2BP1, IGF2BP2, and IGF2BP3) attracted our attention,^30^ with IGF2BP3 ranking fifth overall (Table S1). We then validated these interactions in NPC cells, confirming that IGF2BP1, IGF2BP2, and IGF2BP3 indeed interact with EBNA1 (Fig. 2b). To assess clinical relevance, we analyzed the public transcriptome datasets GSE12452 (NPC GEO dataset) and GSE51575 (Gastric Cancer GEO dataset), and found that, among the IGF2BP family members, only IGF2BP3 exhibited markedly elevated expression in NPC tissues (Fig. 2c) and was highly expressed in gastric cancer tissues alongside IGF2BP1 (Fig. 2d). IP and IF assays further verified the interaction between exogenous EBNA1 (FLAG) and IGF2BP3 (HA) in HK1 and HEK293FT cells (Fig. 2e, f), and importantly, this interaction was confirmed endogenously in EBV-positive C666-1 cells (Fig. 2g, h). Consistent with our findings, Huang’s group reported that IGF2BP3 is the most functionally dominant IGF2BP.^30^ Collectively, these results identify IGF2BP3 as the key EBNA1 interactor worthy of further investigation.

IGF2BP3 contains two N-terminal RNA recognition motifs (RRM1 and RRM2) and four C-terminal hnRNP K homology domains (KH1-4).^31^ To determine which motifs or domains interact with EBNA1, we generated truncated fusion constructs of IGF2BP3-HA (Fig. 2i) and cotransfected them with EBNA1-FLAG plasmids in HEK293FT cells. Our results revealed that EBNA1 bound to the KH3-4 double domain of IGF2BP3 (Fig. 2j, k). Previous studies have reported that the GxxG motif within the KH3-4 double domain is essential for recognizing and binding m^6^A.^30^ Our findings revealed that when the GxxG motif in the KH3-4 double domain was mutated to GEEG, EBNA1 no longer enhanced the binding of IGF2BP3 to the m^6^A-RNA probe, essentially abolishing the interaction between IGF2BP3 and the m^6^A-RNA probe (Fig. 2l).

**Fig. 3:**
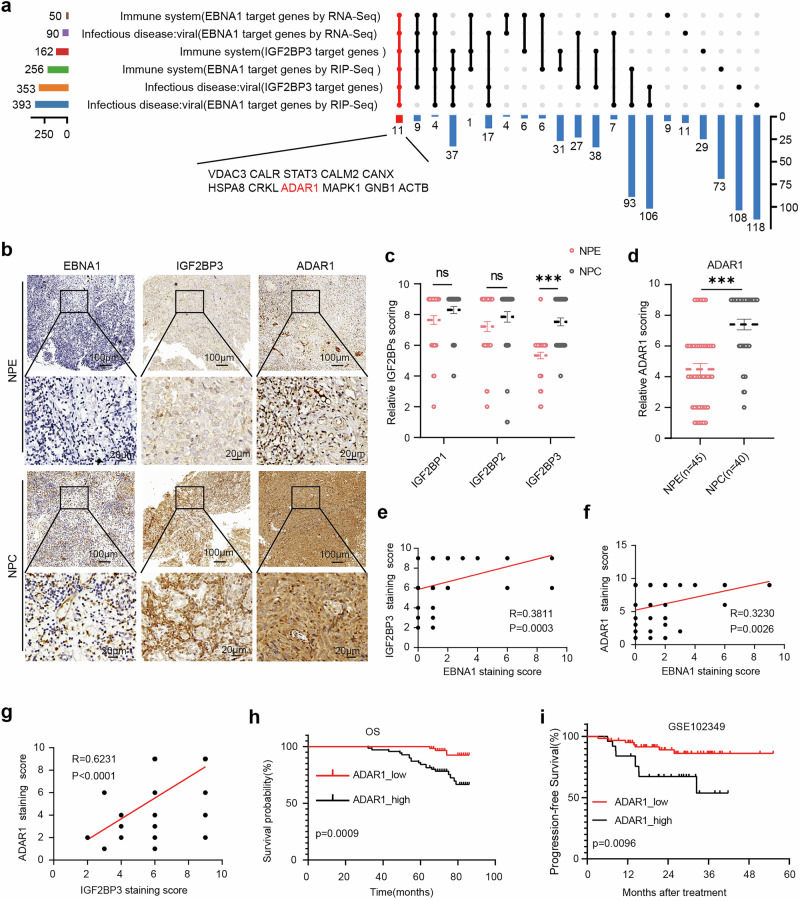
EBNA1 and IGF2BP3 expression positively correlate with ADAR1 expression. **a** Venn analysis was conducted on the three KEGG enrichment results from Supplementary Fig. 2c–e, with a focus on the pathways “immune system” and “infectious disease: viral”, to identify the target gene ADAR1. **b** Representative images of IHC staining for EBNA1, IGF2BP3, and ADAR1 in NPE (n = 45) and NPC (n = 40) samples. Scale bar: 100 µm or 20 µm; magnification: 100× and 400×. **c** Statistical results of IHC staining for IGF2BP1, IGF2BP2, and IGF2BP3 in NPE (n = 45) and NPC (n = 40) samples. **d** Statistical results of IHC for ADAR1 in NPE(n = 45) and NPC(n = 40) samples. **e**‒**g** Pearson correlation analysis of pairwise relationships among EBNA1 expression, IGF2BP3 expression, and ADAR1 expression in 85 clinical samples (40 NPC samples and 45 NPE samples). **h** Kaplan‒Meier analysis of overall survival based on ADAR1 expression levels in 132 NPC patients. **i** Kaplan‒Meier progression-free survival (PFS) analysis of NPC patients with high (black line) or low (red line) ADAR1 expression levels in the GSE102349 dataset. The data were analyzed via two-tailed unpaired t-tests (**c**, **d**), Pearson R statistical tests (**e**–**g**), and log-rank tests (**h**, **i**). ***P < 0.001; ns not significant

To explore whether the EBNA1–IGF2BP3 interaction affects the tumor immune microenvironment, we next analyzed the NPC GEO dataset GSE102349.^32^ This analysis revealed a strong negative correlation between the m^6^A reader IGF2BP3 and interferon pathway-related genes in NPC patients (Supplementary Fig. 2a). Additionally, in the head and neck cancer dataset from TCGA, we observed a negative correlation between IGF2BP3 expression and CD8^+^ T cells infiltration (Supplementary Fig. 2b). These data provide preliminary evidence suggesting that IGF2BP3 may influence tumor immune responses. We subsequently conducted KEGG enrichment analyses on our two datasets, including RNA-Seq target genes from EBNA1-overexpressing tumors (Supplementary Fig. 2c) and EBNA1-RIP-Seq target genes (Supplementary Fig. 2d), to identify gene sets associated with the immune system and viral infection. By integrating these gene sets with the data from Huang et al.‘s study,^30^ which included IGF2BP3-RIP-Seq and CLIP-Seq data, we effectively linked the relevant gene sets of EBNA1 target genes with the immune system and viral infection gene sets of IGF2BP3 target genes (Supplementary Fig. 2e). Venn analyses of the resulting six gene sets revealed 11 predicted target genes, among which adenosine deaminase acting on RNA 1 (ADAR1) attracted our particular attention (Fig. 3a).

ADAR1 has emerged as a key regulator in tumor immunotherapy, with substantial evidence showing that it markedly diminishes tumor responsiveness to ICB treatment.^9–11,33–37^ In this study, we identified a previously unrecognized interaction among the viral protein EBNA1, the m^6^A reader IGF2BP3, and the immune-regulatory gene ADAR1, suggesting that the EBNA1–IGF2BP3 axis may modulate immunotherapy efficacy through ADAR1. To provide preliminary support, immunohistochemistry of a tissue array containing 85 NPC and NPE samples revealed significantly elevated ADAR1 and IGF2BP3 expression in NPC (Fig. 3b–d), whereas the expression of IGF2BP1 and IGF2BP2 was not obviously different (Fig. 3c, Supplementary Fig. 3a). Moreover, both EBNA1 and IGF2BP3 expression was positively correlated with ADAR1 levels (Fig. 3e–g). Analysis of an additional tissue microarray containing 132 NPC cases further demonstrated that high ADAR1 expression was associated with poor overall survival (Fig. 3h). These data underscore the potential importance of these intrinsic molecular connections in the context of NPC progression.^38^

We next integrated multiple public datasets. NPC GSE102349 revealed that elevated ADAR1 expression was significantly associated with diminished progression-free survival in NPC patients (Fig. 3i). NPC GSE12452 revealed a positive correlation trend between ADAR1 and IGF2BP3 expression (Supplementary Fig. 3b). These data were further echoed in the gastric cancer dataset GSE51575, where IGF2BP3 expression also showed a positive association with ADAR1 expression (Supplementary Fig. 3c). Interestingly, this correlation was more pronounced within the EBV-positive subgroup than in the EBV-negative subgroups (Supplementary Fig. 3d), hinting at a possible viral influence on the regulatory process. Building upon these insights, we also examined data from the Cancer Genome Atlas (TCGA) related to head and neck tumors. Our analysis corroborated these previous observations, revealing a consistent positive correlation between IGF2BP3 and ADAR1 expression (Supplementary Fig. 3e), with ADAR1 expression negatively correlated with CD8^+^ T cells infiltration (Supplementary Fig. 3f).

Collectively, these findings suggest that EBNA1 may exert regulatory control over ADAR1 by interacting with the m^6^A reader IGF2BP3, thereby potentially influencing tumor immunosuppression.

### Exogenous EBNA1 enhances ADAR1 translation via IGF2BP3-dependent m^6^A and the recruitment of EIF4G1

**Fig. 4:**
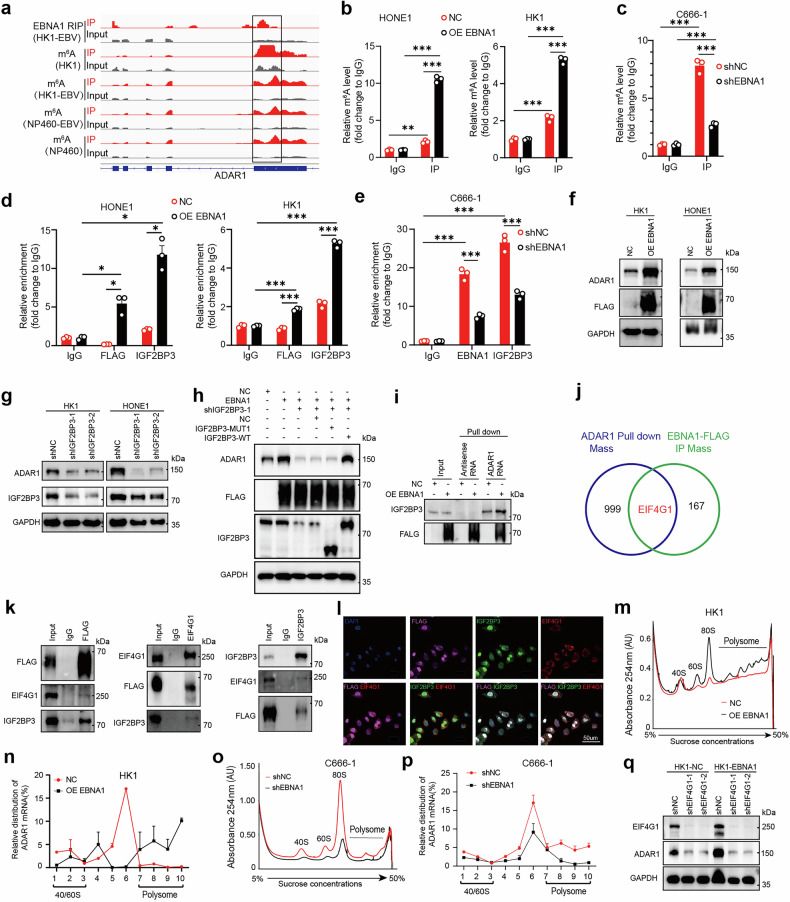
EBNA1 promotes ADAR1 translation but does not affect its mRNA stability. **a** Integrative Genomics Viewer (IGV) tracks displaying the distribution of m^6^A peaks and EBNA1 binding peaks in the ADAR1 transcript. **b** MeRIP experiments were performed using an anti-m^6^A antibody, followed by qPCR analyses in HK1 or HONE1 cells. Rabbit IgG served as a control. The enrichment of the indicated genes was normalized to the input level. **c** MeRIP experiments were performed in C666-1 cells using an anti-m^6^A antibody, followed by qPCR analysis. Rabbit IgG was used as a control. The enrichment of the indicated genes was normalized to the input level. **d** FLAG(EBNA1)-RIP-qPCR and IGF2BP3-RIP-qPCR were performed in HONE1 and HK1 cells (OE EBNA1 group and NC group) to analyze the effect on ADAR1 mRNA enrichment. **e** In C666-1 cells, EBNA1 was knocked down, and EBNA1-RIP-qPCR and IGF2BP3-RIP-qPCR were performed to analyze their effects on ADAR1 mRNA enrichment. **f** After EBNA1 overexpression, Western blotting was used to examine ADAR1 protein levels in NPC cells (HK1, HONE1). **g** After IGF2BP3 was knocked down in NPC cells (HK1, HONE1), ADAR1 protein levels were detected. **h** In EBNA1-overexpressing HEK293FT cells, IGF2BP3 was knocked down followed by reintroduction of IGF2BP3-MUT1 or IGF2BP3-WT, and changes in ADAR1 protein levels were examined. **i** An RNA pull-down assay was used to confirm the enrichment of the IGF2BP3 protein on ADAR1 mRNA. **j** Co-IP and RNA pull-down assays were performed, followed by mass spectrometry, to identify proteins interacting with EBNA1, IGF2BP3, and ADAR1 mRNA, respectively. Venn analysis was conducted to analyze the three groups of proteins. **k** An EBNA1-FLAG plasmid was transfected into HEK293FT cells, and Co-IP was performed using anti-FLAG and anti-EIF4G1 antibodies to isolate the EBNA1-FLAG and EIF4G1 complexes, respectively. Western blotting was used to detect the presence of FLAG (EBNA1) and EIF4G1. **l** IF staining was performed using anti-IGF2BP3, anti-FLAG, and anti-EIF4G1 antibodies to detect the colocalization of exogenous FLAG (EBNA1), endogenous IGF2BP3, and EIF4G1 proteins in EBNA1-overexpressing HK1 cells. Cellular nuclei were stained with DAPI (blue). EIF4G1, FLAG (EBNA1), and IGF2BP3 were visualized in red, pink, and green, respectively. The signals of the two proteins and three proteins were merged, respectively. Scale bar: 50 µm. **m** Sucrose gradient-based polysome profiling analyses were conducted on NC and OE EBNA1 HK1 cells. **n** ADAR1 mRNA in each polysome fraction was quantified via qRT-PCR and plotted as a percentage of the total amount. **o** Sucrose gradient-based polysome profiling analyses were performed on control cells and EBNA1-knockdown cells in C666-1. **p** qRT‒PCR was used to quantify ADAR1 mRNA in each polysome fraction of shNC and shEBNA1 C666-1 cells, and the results were plotted as a percentage of the total amount. **q** Knockdown of EIF4G1 in OE EBNA1 HK1 and NC HK1 cells, followed by Western blot analysis of ADAR1 protein levels. **b**–**q** The results are representative of three independent experiments. **b**–**e** The data are shown as the mean ± s.e.m. Two-way ANOVA with Tukey’s test was used for multiple comparisons. **P < 0.01; ***P < 0.001

The m^6^A reader IGF2BP3 regulates RNA stability by binding to m^6^A-modified transcripts.^30^ Our MeRIP-seq analyses detected m^6^A modifications on ADAR1 mRNA in both NPC and epithelial cells, with the strongest peak overlapping the EBNA1-RIP-seq peak (Fig. 4a), suggesting potential EBNA1 involvement. MeRIP-qPCR revealed that EBNA1 overexpression increased the m^6^A enrichment of ADAR1 mRNA (Fig. 4b), whereas EBNA1 knockdown in EBV-positive C666-1 cells reduced this enrichment (Fig. 4c). RIP-qPCR further demonstrated that both EBNA1 (FLAG) and IGF2BP3 were enriched with ADAR1 mRNA (Fig. 4d), and EBNA1 knockdown markedly impaired this enrichment (Fig. 4e). Collectively, these findings indicate that EBNA1 promotes the recruitment of endogenous IGF2BP3 to m^6^A-modified ADAR1 mRNA (Fig. 4d, e).

To verify whether EBNA1 and IGF2BP3 regulate ADAR1 via m^6^A modification, we designed wild-type (WT) and m^6^A-mutated ADAR1 (Mut) plasmids (Supplementary Fig. 4a) on the basis of the RRACH motif from the EBNA1-RIP-Seq and MeRIP-Seq data in Fig. 4a. In both HK1 and HONE1 cells, the luciferase activity of the WT group was greater than that of the Mut group. Notably, only in the WT group did EBNA1 overexpression result in the highest luciferase activity among all the groups, whereas the activity decreased sharply when IGF2BP3 was knocked down (Supplementary Fig. 4b). In the presence of both EBNA1 and shIGF2BP3, there was no significant difference in firefly luciferase activity between the WT and Mut groups. However, when both exogenous EBNA1 and endogenous IGF2BP3 were present (EBNA1 or EBNA1+shNC), the firefly luciferase activity in the WT group was significantly greater than that in the Mut group (Supplementary Fig. 4b). Similar trends were observed in C666-1 cells, where EBNA1 knockdown reduced WT reporter activity, and additional IGF2BP3 knockdown further suppressed it. Moreover, we observed that, overall, the firefly luciferase activity in the WT group remained significantly greater than that in the Mut group (Supplementary Fig. 4c). Similar results were obtained through RIP‒qPCR analysis of FLAG-EBNA1 and IGF2BP3 enrichment with respect to ADAR1 in HK1 cells. Only in the presence of both EBNA1 and IGF2BP3 did ADAR1 regulation reach its peak (Supplementary Fig. 4d). Moreover, increasing EBNA1 expression progressively increased m⁶A antibody-mediated ADAR1 mRNA enrichment (Supplementary Fig. 4e). Western blot analysis revealed that EBNA1 overexpression increased ADAR1 protein expression across various cell lines (Fig. 4f, Supplementary Fig. 4f).

To move beyond epistatic analysis and pinpoint the exact functional domain required for the EBNA1–IGF2BP3 interaction, we performed rescue experiments in EBNA1-overexpressing cells. After IGF2BP3 knockdown, reintroduction of IGF2BP3-WT partially restored the activity of the ADAR1-WT reporter and fully restored ADAR1 protein expression (Supplementary Fig. 4i, Fig. 4h), whereas the KH3–KH4 domain-deficient mutant (IGF2BP3-MUT1) failed to do so (Supplementary Fig. 4i, Fig. 4h). These findings indicate that the KH3–KH4 domains are essential for IGF2BP3 function. Furthermore, RNA pull-down experiments revealed that ADAR1 mRNA was enriched with the IGF2BP3 protein, and this enrichment increased upon EBNA1 overexpression (Fig. 4i). These data suggest that EBNA1 requires IGF2BP3 to regulate ADAR1, with IGF2BP3 acting as a bridge gene.

Previous studies have shown that IGF2BP family proteins increase mRNA stability and translation in a m^6^A-dependent manner.^30,31^ We first examined whether EBNA1 affects ADAR1 mRNA stability, but no significant changes were observed, indicating that EBNA1 does not regulate ADAR1 through RNA stabilization (Supplementary Fig. 4j). We therefore explored alternative mechanisms. Notably, through IP-MS and RNA pull-down MS, we identified eukaryotic translation initiation factor 4G1 (EIF4G1), a crucial component of the ribosomal translation machinery,^39^ as a top-ranked protein that interacts with EBNA1, IGF2BP3, and ADAR1 mRNAs (Fig. 4j). These findings suggest that EBNA1 influences ADAR1 translation. Co-IP and immunofluorescence experiments confirmed the interaction between EBNA1, IGF2BP3, and EIF4G1(Fig. 4k, l).

Polysome profiling revealed that EBNA1 overexpression increased polysome abundance and enriched ADAR1 mRNA within polysome fractions, indicating enhanced translation (Fig. 4m, n; Supplementary Fig. 4k, l). Conversely, EBNA1 knockdown in C666-1 cells reduced polysome levels and decreased ADAR1 mRNA association with polysomes (Fig. 4o, p). Silencing EIF4G1 nearly eliminated ADAR1 protein expression (Fig. 4q). These data demonstrate that EBNA1 promotes ADAR1 translation.

Taken together, EBNA1 and IGF2BP3 promote ADAR1 translation by recruiting EIF4G1, forming a key translational regulatory complex that enhances ADAR1 protein synthesis.

### EBNA1-mediated ARDR1 reduces interferon release and is associated with diminished tumor responsiveness to immunotherapy

ADAR1 editing of dsRNA can neutralize the immunogenicity of endogenous dsRNAs, reduce interferon pathway activation, and suppress immune responses, ultimately contributing to resistance to ICB therapy.^9^ Given that ADAR1 is regulated by EBNA1, we investigated whether EBNA1 influences the role of ADAR1 in the tumor immune environment or its impact on resistance to immunotherapy.

**Fig. 5:**
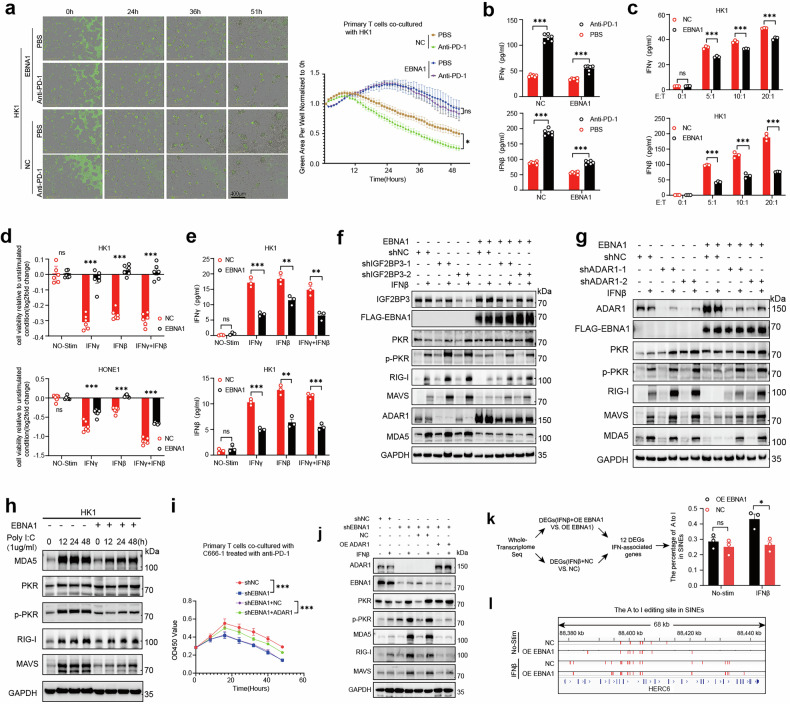
The presence of EBNA1 reduces tumor responsiveness to immunotherapy. **a** Control cells (HK1-NC) and EBNA1-overexpressing cells (HK1-EBNA1) were cocultured with CD8^+^T cells, respectively. The immunotherapy effect on both groups of tumor cells was evaluated. **b** The secretion levels of IFNγ and IFNβ were measured in the cell supernatants shown in (**a**). **c** At effector-to-target (E:T) ratios of 0:1, 5:1, 10:1, and 20:1, EBNA1-overexpressing HK1 cells and control cells were cocultured with CD8^+^T lymphocytes. The secretion levels of IFNβ and IFNγ were measured. **d** After stimulation with IFNγ, IFNβ, or a combination of IFNγ + IFNβ, the growth and viability of OE EBNA1 HK1 cells and NC HK1 cells were assessed. **e** ELISA was used to measure the secretion levels of IFNβ and IFNγ in the culture supernatants of NPC cells (EBNA1 HK1 and NC HK1) under the following conditions: Unstimulated, IFNβ-stimulated, IFNγ-stimulated, and combined IFNβ + IFNγ-stimulated. **f** EBNA1-overexpressing or IGF2BP3 knockdown HK1 cells were stimulated with IFNβ, and western blotting was used to detect the protein expression levels of ADAR1 and downstream RNA sensor molecules. **g** EBNA1-overexpressing or ADAR1 knockdown HK1 cells were stimulated with IFNβ, and western blotting was used to detect changes in downstream RNA sensor molecules. **h** Poly I:C was used to treat EBNA1-overexpressing HK1 cells at different time points, and western blotting was performed to detect changes in the following proteins: MDA5, PKR, RIG-I, pPKR (phosphorylated PKR), and MAVS. **i** C666-1 cells with EBNA1 knockdown were reintroduced with ADAR1 and cocultured with CD8⁺ T cells (effector-to-target ratio = 20:1). The immunotherapy effects on four groups of tumor cells (shNC, shEBNA1, shEBNA1+NC, and shEBNA1 + ADAR1) were evaluated using the CCK8 assay. **j** In C666-1 cells, after EBNA1 knockdown followed by ADAR1 reintroduction, Western blot analysis was performed to examine the changes in the expression of RNA sensors (MDA5, MAVS, pPKR, and RIG-I) after interferon stimulation. **k** Differentially expressed genes in EBNA1-overexpressing cells after IFNβ stimulation and differentially expressed genes in control tumor cells after IFNβ stimulation were subjected to Venn analysis, yielding 96 interferon-related genes. Among these genes, 12 interferon-related genes (UBE2L6, SP100, IFIH1, EIF2AK2, LGALS9, CMPK2, IDO1, DHX58, HERC6, HSH2D, IFIT3, and OAS3) of interest were selected for A-to-I analysis (n = 3 for each condition). **l** In whole-transcriptome sequencing, A-to-I editing changes were detected in the SINE region of HK1 cells under conditions with or without IFN stimulation and with or without EBNA1 expression. These changes were then mapped to specific transcript locations (red lines). **a**–**e**, **i** The results are shown as the means ± s.e.m.s. Two-tailed unpaired t-test. *P < 0.05; **P < 0.01; ***P < 0. 00; ns, not significant. The results are representative of three independent experiments

We initially cocultured tumor cells (labeled with GFP) with T cells in vitro and utilized the Sartorius Incucyte® SX5 live-cell analysis system to monitor the effects of immunotherapy. The results revealed that after the administration of an anti-PD-1 antibody, T cells were less effective at eliminating EBNA1-overexpressing tumor cells than were control cells (Fig. 5a). Additionally, we assessed interferon levels in the supernatants of cocultured cells. Both IFNγ and IFNβ levels were increased after anti-PD-1 treatment, but the increase was greater in control cells than in EBNA1-overexpressing tumor cells (Fig. 5b). In another experiment, primary T cells were cocultured with HK1-EBNA1 or control tumor cells at various effector:target (E: T) ratios (0:1, 5:1, 10:1, and 20:1) for 43 h. We observed that the secretion levels of IFNγ and IFNβ were significantly lower in HK1-EBNA1 cells than in control cells (Fig. 5c). These data suggest that EBNA1 can reduce the capacity of T cells to eliminate cancer cells by diminishing interferon production and the response to PD-1 therapy.

Since interferons are known to bypass CD8^+^ T-cell recognition to exert therapeutic effects,^9^ we treated tumor cells directly with interferons (IFNγ, IFNβ, and both) and assessed the viability of EBNA1-overexpressing HK1 or HONE1 cells and control cells. We observed that interferon-mediated inhibition was also less effective in EBNA1-overexpressing tumor cells than in control cells, even without T-cell involvement (Fig. 5d). Notably, without interferon stimulation, neither EBNA1-overexpressing nor control cells released detectable amounts of IFNγ or IFNβ. However, upon stimulation, EBNA1-overexpressing tumor cells released fewer interferons than control cells did (Fig. 5e). These results indicate that exogenous EBNA1 can suppress the response to interferon, potentially by decreasing autocrine interferon release.

To further investigate the impact of EBNA1–IGF2BP3 on ADAR1-mediated RNA sensor activity, we examined the effect of depleting IGF2BP3, which interacts with EBNA1, on the expression of RNA sensors upon interferon stimulation. We observed that treating tumor cells with IFNβ generally increased the expression levels of several RNA sensors (MDA5, MAVS, pPKR, and RIG-I) to varying degrees. Interestingly, in tumor cells lacking EBNA1, depleting IGF2BP3 after IFNβ stimulation resulted in a relative increase in the expression of these RNA sensors to varying degrees compared with that in tumor cells overexpressing EBNA1 (Fig. 5f). Additionally, in both EBNA1-overexpressing and control tumor cells, the knockdown of ADAR1 following IFNβ treatment led to varying degrees of increased expression of RNA sensors (MDA5, MAVS, pPKR, and RIG-I), but these changes were relatively minor in EBNA1-overexpressing cells (Fig. 5g). To investigate how EBNA1 mediates ADAR1 editing of dsRNA and its subsequent effect on dsRNA sensors, we also utilized poly I:C, a synthetic analog of dsRNA,^40^ to stimulate tumor cells. Notably, we observed that poly I:C stimulation did not significantly alter dsRNA sensor levels in EBNA1-overexpressing tumor cells but did increase dsRNA sensor levels in control cells (Fig. 5h).

To validate the regulatory role of the EBNA1–ADAR1 axis under physiological conditions, we knocked down EBNA1 in C666-1 cells and then reintroduced ADAR1. These treated cells were cocultured with T cells and subjected to PD-1 therapy. We observed that EBNA1 knockdown enhanced T-cell cytotoxicity in C666-1 cells, whereas ADAR1 reintroduction attenuated T-cell killing of tumor cells (Fig. 5i). After interferon stimulation, the protein expression of RNA sensors (MDA5, MAVS, pPKR, and RIG-I) was markedly increased in the EBNA1-knockdown group, whereas this increase was substantially diminished in the ADAR1 reintroduction group (Fig. 5j).

Finally, we utilized whole-RNA sequencing to examine EBNA1-mediated ADAR1 A-to-I editing of dsRNAs in small interspersed nuclear elements (SINEs). Considering the established connections between EBNA1 and ADAR1 interactions and their roles in interferon pathways and tumor immunity, we focused on SINEs containing high levels of A-to-I editing events, particularly those adjacent to interferon-associated genes. Under the amplified effect of interferon stimulation, a significant increase in the level of A-to-I edits within SINEs associated with approximately 12 differentially expressed interferon-associated genes was observed in EBNA1-expressing cells compared with control cells (Fig. 5k). Moreover, in EBNA1-expressing cells, the number of A-to-I RNA-editing sites was significantly greater upon IFNβ induction than in the other control groups (Fig. 5l, Supplementary Fig. 4m), despite unchanged ADAR1 mRNA levels (Supplementary Fig. 4n). These data were corroborated by Sanger sequencing, which confirmed increased A-to-G changes in specific SINE loci in EBNA1-expressing cells upon IFNβ stimulation (Supplementary Fig. 4o). Together, these findings suggest that EBNA1 enhances the frequency of ADAR1-mediated A-to-I editing events following interferon induction. This may reduce the presence of unedited dsRNAs (non-self) that could serve as ligands for dsRNA sensors.

Collectively, our data indicate that exogenous EBNA1 enhances ADAR1 A-to-I editing by dsRNAs, leading to suppressed RNA sensor activity and reduced interferon release. This confers resistance to the efficacy of immunotherapy in vitro.

### Targeting EBNA1 with a PROTAC degrader enhances the efficacy of ICB therapy in CD34^+^ humanized mice

To validate our findings, we developed a PROTAC degrader that targets EBV-EBNA1 and conducted both in vitro and in vivo experiments.

**Fig. 6:**
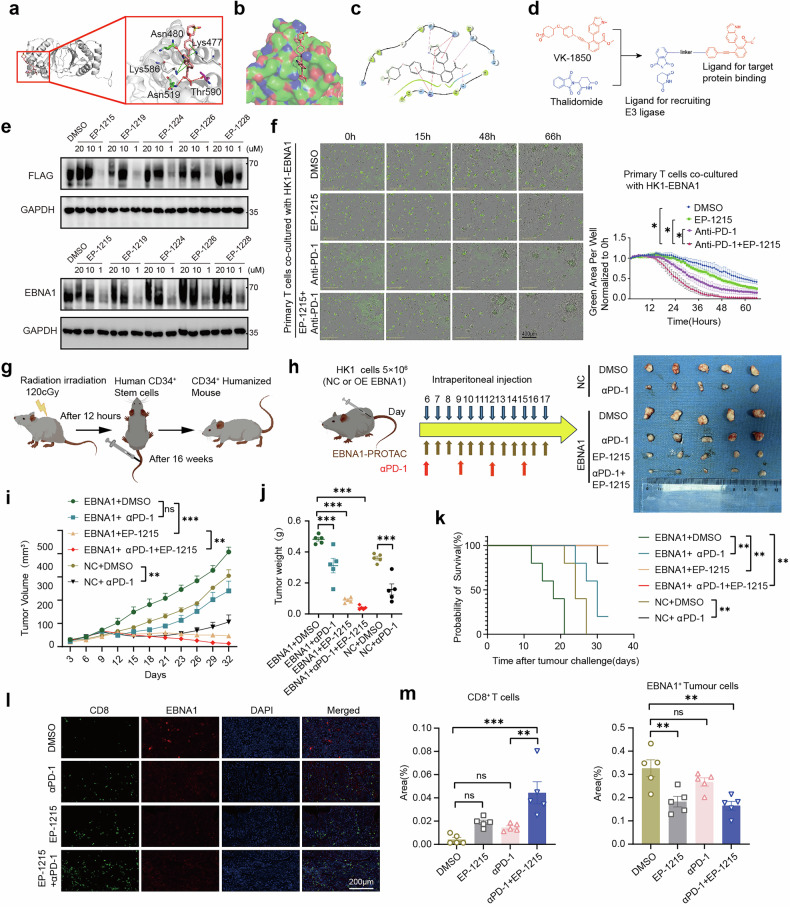
EP-1215 enhances the efficacy of immune checkpoint inhibitors in humanized CD34^+^ mouse models. **a** Docking pose of VK-1850 within the catalytic pocket of EBNA1 (PDB: 6NPP). **b** The binding mode of VK-1850 in the EBNA1 catalytic pocket. **c** Two-dimensional docking fitting diagram of VK-1850 at the EBNA1 binding site. **d** Design strategy for EBNA1 PROTACs. **e** HEK293FT cells overexpressing EBNA1-FLAG or EBNA1 were treated with five different EBNA1 degraders at varying concentrations for 72 h, and the degradation levels were assessed via western blotting. **f** EBNA1(labeled with GFP)-overexpressing HK1 cells were cocultured with T cells, and the therapeutic effects of EP-1215, αPD-1 monotherapy, or a combination of EP-1215 and αPD-1 treatment were evaluated. **g** Workflow for establishing the CD34^+^ humanized mouse model. The mouse diagram was created via FigDraw. **h** Xenograft tumors were established with HK1 control and EBNA1-overexpressing HK1 cells in CD34^+^ humanized mice. The mice were treated with DMSO, αPD-1, EP-1215, or αPD-1 combined with EP-1215. The mouse diagram was created via FigDraw. Representative images of xenograft tumors from different treatment groups are shown (n = 5 mice per group). **i**, **j** Tumor volume and tumor weight of the mice shown in (**g**) (n = 5 mice per group). **k** Survival analyses of the mice shown in (**g**) (n = 5 mice per group). **l**, **m** Representative IF images showing the characteristics of CD8^+^ T cells (green) and EBNA1 expression (red) in tumor cells following αPD-1 monotherapy or a combination of αPD-1 and EP-1215 treatment. The cell nuclei were stained with DAPI (blue). Scale bar: 200 µm. (n = 5 mice per group). **f**, **i** Data are shown as the mean ± s.e.m. **f**, **i**, **j**, **m** Two-way ANOVA with Tukey’s test for multiple comparisons. **k** Log-rank test. *P < 0.05;**P < 0.01; ***P < 0.001; ns, not significant. **e**, **f** Results are representative of three independent experiments

We synthesized five candidate PROTAC degraders on the basis of a previous study^14^ (Fig. 6a–d) and observed that all five degraders effectively degraded EBNA1 at a concentration of 1 μM. However, the degradation effect was abolished at higher concentrations of 10 μM and 20 μM (Fig. 6e). This may be attributed to the “hook effect” of PROTACs, where high concentrations tend to form nonfunctional binary complexes (PROTAC:ligase or PROTAC:POI) rather than the ternary complex required for effective degradation. These results suggest that PROTACs are efficient degraders capable of minimizing cellular side effects.^41^ Interestingly, we next compared one of our degraders, EP-1215, with the reported EBNA1 inhibitor VK-1727.^14^ As expected, VK-1727 did not degrade EBNA1, whereas EP-1215 effectively degraded EBNA1 and reduced ADAR1 protein levels (Supplementary Fig. 5a). Consistently, we also confirmed that EP-1215 could degrade EBNA1 and lead to a decrease in the ADAR1 protein in C666-1 cells (Supplementary Fig. 5b). Furthermore, we examined the in vitro effects of EP-1215, an anti-PD-1 antibody, and their combination on T-cell-mediated tumor cell killing. Notably, combined treatment with EP-1215 and an anti-PD-1 antibody significantly outperformed either treatment alone (Fig. 6f).

To better validate the potency of our degrader, we utilized a C57BL/6 mouse model and the B16 melanoma cell line. Mice were engrafted with EBNA1-overexpressing B16 cells or control cells, followed by treatment with DMSO, an anti-PD-1 antibody, EP-1215, or a combination of PD-1 and EP-1215. In the EBNA1-overexpressing B16 cell group, neither EP-1215 nor the PD-1 antibody alone demonstrated obvious therapeutic efficacy, but their combined application yielded a strong therapeutic effect. Conversely, in the control group harboring B16 cells without EBNA1 overexpression, the PD-1 antibody alone was effective (Supplementary Fig. 5c–f). Flow cytometry analysis revealed that, in the EBNA1-overexpressing B16 cell group, the combination of EP-1215 and an anti-PD-1 antibody elicited the highest level of CD8^+^ T cells infiltration and a concomitant increase in IFNγ^+^ CD8^+^ T cells (Supplementary Fig. 5g, h). In the monotherapy groups treated with either PD-1 or EP-1215, CD8^+^ T-cell infiltration and IFNγ^+^ CD8^+^ T-cell levels were similar to those in the DMSO-treated group (Supplementary Fig. 5g, h). Notably, in the control B16 cell group, PD-1 antibody treatment increased both CD8^+^ T-cell infiltration and IFNγ^+^CD8^+^ T-cell infiltration relative to those in the DMSO-treated group (Supplementary Fig. 5g, h).

To further investigate whether EP-1215 could enhance the efficacy of PD-1 antibody therapy in human tumors, we established a CD34^+^ humanized mouse model (Fig. 6g). Mice with more than 20% CD45^+^ cells in their peripheral blood were selected for inclusion in the humanized mouse study (Supplementary Fig. 5i). Mice bearing NPC xenografts derived from HK1 cells overexpressing EBNA1 were treated with DMSO, PD-1 antibody, EP-1215, or a combination of EP-1215 and PD-1 antibody. Control HK1 xenografts were treated with either DMSO or an anti-PD-1 antibody. Notably, both EP-1215 and the PD-1 antibody effectively inhibited tumor growth in the EBNA1-overexpressing HK1 xenografts (Fig. 6h–k), and combined treatment with EP-1215 and the PD-1 antibody had a significantly greater inhibitory effect. Furthermore, PD-1 antibody treatment alone was effective in controlling HK1 xenograft tumors, with a more pronounced effect than PD-1 antibody treatment in the EBNA1-overexpressing group. These findings suggest that EBNA1 can partially enhance tumor resistance to immunotherapy in the tumor immune microenvironment. (Fig. 6i–k). Multiplex IF analysis confirmed that CD8^+^ T cells infiltrated the tumor tissue more extensively in the EP-1215 and PD-1 antibody-treated groups than in the DMSO-treated group, with the combination of EP-1215 and PD-1 antibody resulting in the highest level of CD8^+^ T cells infiltration (Fig. 6l, m).

We also evaluated the safety of EP-1215 by assessing liver and kidney function through serum markers (Supplementary Fig. 5j, k) and conducting HE staining (Supplementary Fig. 5l). We did not observe any abnormal changes in liver or kidney biomarkers in the serum of EP-1215-treated mice, nor did we observe significant infiltration of inflammatory cells or tissue necrosis via HE staining compared with the DMSO group. These results indicated that EP-1215 did not induce observable hepatotoxicity or nephrotoxicity, suggesting a preliminary tolerability that warrants further safety profiling.

Finally, we investigated the efficacy of EP-1215 in EBV-positive models. In vitro, T cells coculture with C666-1 cells revealed that the combination of EP-1215 and anti-PD-1 induced the most robust cytotoxicity, and EP-1215 alone also enhanced killing compared with the PBS control (Supplementary Fig. 5m). In PBMC-humanized mice bearing C666-1 xenografts, EP-1215 alone exhibited significant antitumor efficacy, which was further enhanced upon combination with anti-PD-1, resulting in a marked reduction in tumor volume (Supplementary Fig. 5n).

Collectively, these data indicate that EP-1215 achieves its antitumor effects by degrading EBNA1 and consequently sensitizing tumors to immunotherapy.

## Discussion

**Fig. 7:**
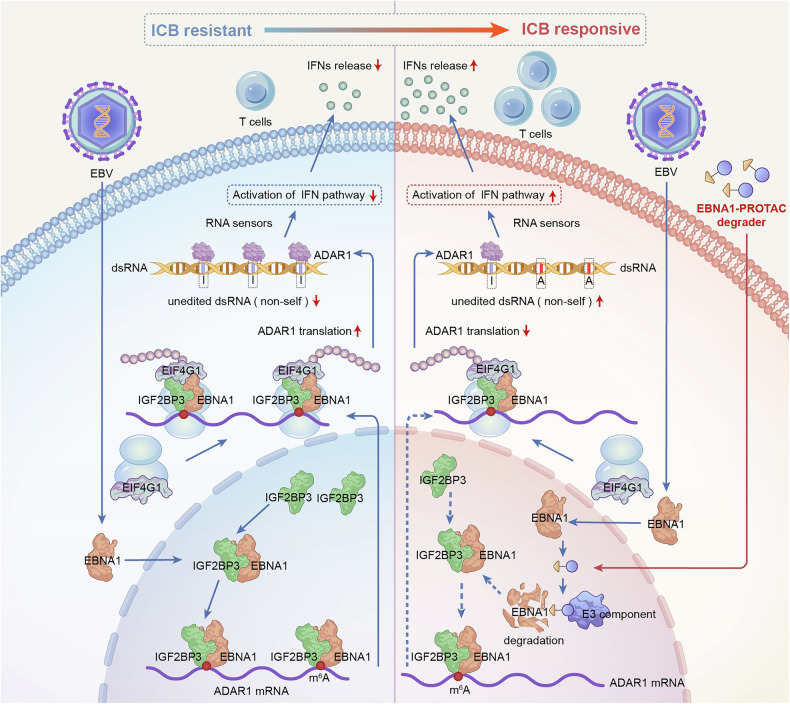
Working model. EBV-EBNA1 interacts with IGF2BP3 in the nucleus and recognizes ADAR1 mRNA through m^6^A modifications. EBNA1 subsequently recruits the translation initiation factor EIF4G1, increasing the ribosomal translation efficiency of ADAR1 mRNA. As a result, increased levels of ADAR1 protein lead to increased editing of A-to-I in dsRNA, reducing non-self dsRNA. This reduction weakens the ability of downstream RNA sensors to recognize dsRNA, causing interferon pathway inactivity and decreased interferon release, ultimately decreasing the sensitivity of tumor cells to immunotherapy. When we added the EBNA1-PROTAC degrader, EBNA1 was degraded, which reduced the binding of IGF2BP3 to ADAR1 mRNA. This further decreases the recruitment of EIF4G and the translation efficiency of ADAR1, leading to a decrease in ADAR1 protein levels. This results in reduced A-to-I editing of dsRNA, increasing non-self dsRNA, thereby activating the interferon pathway and IFN release, ultimately increasing the sensitivity of tumor cells to immunotherapy. The schematic illustration was generated using Adobe Illustrator

In the ongoing quest to unravel the complexities of human health and disease, EBV, as the first identified oncogenic virus, remains a particularly intriguing puzzle.^42^ It has long been acknowledged that EBV is closely associated with several malignancies.^14,43,44^ This study represents an in-depth investigation into the function of the core antigen of EBV, EBNA1, which acts as an exogenous regulator in shaping the tumor immunosuppressive microenvironment through its modulation of ADAR1, a crucial enzyme involved in dsRNA editing processes. By revealing this previously unknown mechanism, our research underscores the ability of EBNA1 to reshape the immunological landscape and lead to tumor resistance to immunotherapy (Fig. 7).

We began by functionally characterizing the immunosuppressive role of EBNA1 in vivo. Single-cell sequencing data revealed that the overexpression of EBNA1 in NPC cells led to a substantial decrease in CD8^+^ T-cell infiltration and natural killer (NK) cell activity, coupled with an increase in the number of immunosuppressive M2 macrophages. This shift toward an immunosuppressive environment is characterized by a substantial reduction in proinflammatory cytokines such as IFNγ and IFNβ, which are essential for effective cytotoxic T lymphocyte responses. The suppression of this inflammatory milieu impairs the efficacy of ICB therapy, underscoring the specific role of EBNA1 in establishing a tumor microenvironment conducive to tumor progression and immune suppression. Additionally, single-cell sequencing revealed that these tumor CD8^+^ T cells undergo G1-phase cell cycle arrest, further diminishing their antitumor activity. These observations not only align with previous studies on EBV-associated malignancies^45^ but also provide mechanistic insights by directly linking these immune changes to the activity of EBNA1.

### Our study yielded several key discoveries

1. Identification of ADAR1 as an unexplored host target of viral proteins. ADAR1, a central focus in tumor immunotherapy research,^46–48^ has been identified as a target of the interaction between exogenous EBNA1 and IGF2BP3. This discovery paves the way for insights into virus-associated gene regulatory mechanisms and holds promising implications for therapeutic interventions targeting RNA-editing processes.
2. The role of eIF4G1 in the initiation of ADAR1 translation. EIF4G1, an important scaffolding protein of the eIF4F complex, plays a crucial role in binding to other translation initiation factors, facilitating the efficient translation of mRNAs and thereby participating in the initiation phase of protein synthesis.^49^ Our study revealed that exogenous EBNA1 can form a complex with the m^6^A reader IGF2BP3 and the translation initiation factor EIF4G1. The formation of this complex enhances the translation efficiency of ADAR1, shedding light on previously unexplored layers of ADAR1 expression regulation. To further validate the function of the EBNA1–IGF2BP3 interaction, we employed a strategic genetic approach. Facing technical challenges in generating EBNA1 truncation mutants, we precisely disrupted the interaction interface by mutating the KH3–KH4 domains of IGF2BP3. This intervention completely abolished the ability of EBNA1 to upregulate ADAR1, providing decisive evidence for the critical nature of this interaction and effectively creating a functional proxy for an interaction-defective EBNA1 mutant.
3. Viral suppression of immunity via ADAR1-edited dsRNA. We demonstrated that the EBNA1–IGF2BP3-EIF4G1 axis drives enhanced A-to-I editing of dsRNAs within SINE elements near interferon genes, which is consistent with the substrate preference of ADAR1 for noncoding regions and its established link to interferon signaling.^50–52^ The observed increase in A-to-I editing, coupled with the suppression of dsRNA sensor activation, aligns with the prevailing “structural defusing” model in which I:U mismatches disrupt perfect dsRNA duplexes, leading to their failure to activate cytosolic sensors such as MDA5 and PKR, which require stable duplex structures for activation.^9,47^ These findings provide a plausible mechanistic basis for the observed interferon pathway inhibition in our experimental system. Future structural studies will be crucial to obtain direct, high-resolution evidence of these structural modifications in the specific context of EBNA1-mediated immunosuppression. In addition to host RNA editing, we propose that ADAR1 upregulated via this axis may also edit EBV-encoded RNAs during other infection phases (e.g., the lytic cycle), potentially masking viral dsRNA or stabilizing viral transcripts to further enhance immune evasion. Systematic mapping of the EBV ‘dsRNA editome’ across different infection states, therefore, represents a promising direction for future research.^53^

By targeting this axis, we developed a first-in-class EBNA1-targeted PROTAC degrader (EP-1215), which can effectively degrade the EBNA1 protein. This approach represents an advance in the development of therapeutics for EBV-associated malignancies such as NPC, as no drugs have been validated thus far in vivo for directly degrading the viral protein EBNA1 and alleviating tumor immunosuppression, despite existing reports on the development and function of EBNA1 inhibitors.^14^ Notably, our study demonstrated that EP-1215, in combination with PD-1 blockade, had a highly synergistic effect, leading to increased CD8^+^ T-cell infiltration and increased IFNγ production in tumors. Compared with either treatment alone, this combination therapy significantly improved antitumor efficacy, suggesting its potential as a strategy for overcoming resistance to ICB therapies in EBV-associated malignancies. Notably, the humanized mouse model used in this study allowed us to validate the therapeutic potential of EP-1215 in a system that closely mimics human immune responses. A recent report in which siADAR1 was used to silence ADAR1 expression and mobilize synergistic antitumor immunity showed notable effectiveness,^54^ highlighting the potential of targeted ADAR1-mediated promotion of tumor immunotherapy. Interestingly, our strategy precisely targets and degrades the viral protein EBNA1, thereby safely modulating ADAR1 within tumor tissues without harming normal tissue cells. This approach demonstrates practicality and a favorable safety profile.^55–57^ Indeed, our safety data indicate that EP-1215 does not induce hepatotoxicity or nephrotoxicity, underscoring its potential for clinical translation. Therefore, we are planning to encapsulate our PROTAC EBNA1 degrader via a novel exosome-based droplet technology,^58^ formulating it as a nasal spray for clinical application in combination with PD-1 antibodies, which should be of interest to clinicians.

We acknowledge two primary limitations that guide future work. First, the technical challenges of sequencing (e.g., Sanger sequencing), and highly repetitive SINE regions limit comprehensive orthogonal validation of RNA-editing sites.^51,59^ Although we successfully validated a subset of sites, broader and more efficient methods for probing RNA editing, even in clinical samples, will be essential to fully elucidate its functional impact. Second, a full structure‒function analysis of EBNA1 is needed to definitively map its binding interface with IGF2BP3, building on our current approach. The creation of such specific EBNA1-defective mutants would inspire future studies to unequivocally delineate the functional contribution of this particular interaction to immunosuppression, disentangling it from the other multifaceted roles of EBNA1 in oncogenesis. This represents both a challenge and an exciting direction for future research.

In conclusion, the implications of our findings extend beyond NPC to other EBV-driven malignancies. The EBNA1–IGF2BP3-EIF4G1-ADAR1 axis likely represents a broader viral strategy for tumor immune suppression, suggesting that targeting this axis could enhance immunotherapy responses across a range of EBV-positive malignancies. Given the critical role of ADAR1 in regulating RNA-sensing pathways and immune checkpoints, therapies targeting this axis, particularly EBNA1, could provide a useful approach for sensitizing tumors to ICB treatments. Thus, this study provides insights into the immune-suppression mechanisms involved in EBV-associated malignancies and suggests potential therapeutic avenues for treating EBV-positive malignancies by elucidating the EBNA1–IGF2BP3-EIF4G1-ADAR1 axis and developing EBNA1-targeting PROTACs.

## Materials and methods

### Ethics statement

Our study, involving the use of human tissues, adhered strictly to all relevant ethical guidelines approved by the Medical Ethics Committee at Southern Medical University (SMU). Informed consent was obtained from each patient prior to the collection of any clinical samples. Furthermore, the welfare and treatment of the experimental animals were conducted in accordance with the standards outlined in the National Institutes of Health’s Guide for the Care and Use of Laboratory Animals. All protocols related to animal care and use were reviewed and approved by the Ethics Committee of Shenzhen Hospital affiliated with SMU under reference No. 2021–0132.

### Patient samples

Two distinct tissue arrays were employed. The first array comprised samples from 40 NPC patients and 45 normal controls with chronic rhinitis provided by Shenzhen Hospital, affiliated with SMU. These samples were subjected to rigorous clinical diagnosis by three experienced pathologists. The second array featured 132 NPC specimens accompanied by corresponding survival data sourced from Shanghai Outdo Biotech. The utilization of these human tissues was formally authorized by the Medical Ethics Committee of SMU, ensuring adherence to ethical standards (Ref. No. SZYYEC2020R001).

### Cell Culture

The cell lines used in this study included the nasopharyngeal epithelial cell lines NP460 (hTert-immortalized) and EBV-positive NP460-EBV. These were cultured in a 1:1 mixture of D-KSFM (Gibco,10744019) complete medium and EpiLife medium containing EpiLife defined growth supplement (EDGS) (Gibco, MEPI500CA) under serum-free conditions. The NPC cell lines HK1, SUNE1, and HONE1 and the EBV-positive NPC cell lines HK1-EBV and C666-1 were cultured in RPMI 1640 medium (HyClone, SH30027. FS) supplemented with 10% fetal bovine serum (FBS; Gibco, 10099141). Mouse pregastric cancer cells (MFCs), B16 melanoma cells (B16), and HEK293FT cells were cultured in high-glucose DMEM (HyClone, SH30243.01) supplemented with 10% FBS. All media were supplemented with 1% penicillin‒streptomycin, and the cells were maintained at 37 °C in a 5% CO₂ humidified incubator. All the cells were subjected to mycoplasma testing (Vazyme, D101-01) and short tandem repeat analyses. The NP460, NP460-EBV, HK1, HK1-EBV, and C666-1 cell lines were generous gifts from Professor George Sai Wah Tsao, University of Hong Kong. The SUNE1 cell line was kindly provided by Professor Na Liu from Sun Yat-sen University Cancer Center.^60^ The HONE1 cell line was generously provided by Professor Wei Xiong from Central South University.^61^ The MFC cell line was a gift from Professor Bingxia Zhao, Southern Medical University. The B16 and HEK293FT cell lines were obtained from our laboratory.

### CD34^+^ humanized NCG mice

NOD/ShiLtJGpt-Prkdc^em26Cd52^Il2rg^em26Cd22^/Gpt (NCG) mice were used for the production of humanized NCG mice. Four-week-old NCG mice were preconditioned by whole-body sublethal irradiation (120 cGy) at least 6 h prior to intravenous injection of CD34^+^ HSCs (from Oibiotech). To facilitate accurate identification and easy access to the tail veins of preconditioned NCG mice, the mice were exposed to infrared light to induce vasodilation. Subsequently, 10,000 CD34^+^ HSCs resuspended in 200 µL of PBS were intravenously injected into each preconditioned NCG mouse via a 1-mL insulin syringe with a detachable 26G needle. After transplantation of CD34^+^ hematopoietic stem cells, the percentage of human CD45^+^ cells in CD34^+^ humanized mice was monitored at week 16 by flow cytometry. CD34^+^ humanized mice with more than 20% human CD45^+^ cells in the blood were used for the establishment of xenografts.

### Animal experiments

The mice used for tumor inoculation were aged between 4 and 8 weeks. For the nontreatment experiments, 1.0 × 10^6^ B16 cells were subcutaneously injected into C57BL/6 mice, and the tumor growth results are shown in Fig. 1c. For the tumor inoculation experiments in NCG mice, 5.0 × 10^6^ HK1 cells were subcutaneously injected, and the tumor growth results are shown in Fig. 1b. For the drug treatment experiments, involving tumor inoculation in C57BL/6 mice, 1.0 × 10^5^ B16 cells were subcutaneously injected, and the mice were intraperitoneally injected with 200 μg of InVivoMAb anti-mouse PD-1 (Bio X Cell, clone: RMP1-14) on days 9, 11, 13, and 15. The mice were intraperitoneally injected daily with 60 mg/kg of EBNA1-PROTAC (EP-1215) from days 9 to 16. The detailed grouping results are shown in Supplementary Fig. 5c. In CD34^+^ humanized NCG mice, 5.0 × 10^6^ HK1 cells were subcutaneously injected, and mice were intraperitoneally injected with 200 μg of InVivoMAb anti-human PD-1 (Bio X Cell, clone: J116) on days 6, 9, 12, and 15. Mice were intraperitoneally injected daily with 60 mg/kg of EBNA1-PROTAC (EP-1215) from days 6 to 17. The detailed grouping results are shown in Fig. 6h. In PBMC-humanized NCG mice, 1.0 × 10^7^ C666-1 cells were inoculated subcutaneously, and 200 μg of InvivoMAb anti-human PD-1 antibody (Bio X Cell, clone: J116) was administered intraperitoneally on days 6, 9, 12, and 15. From day 6 to day 20, the mice were intraperitoneally injected daily with 60 mg/kg EBNA1-PROTAC (EP-1215). Tumor growth was monitored every two or three days, and the tumor volume was calculated via the formula (length × width²)/2.

### Single-cell RNA sequencing (scRNA-seq) and data analysis

The cell suspension was loaded into chromium microfluidic chips with 3′ v3.1 chemistry and barcoded with a 10× chromium controller (10× Genomics). RNA from the barcoded cells was subsequently reverse transcribed, and sequencing libraries were constructed with reagents from a Chromium Single Cell 3′ v3.1 reagent kit (10×Genomics) according to the manufacturer’s instructions. Sequencing was performed with an Illumina NovaSeq 6000 according to the manufacturer’s instructions (Illumina).

Raw FASTQ files were processed via Cell Ranger (v7.1.0, 10× Genomics). Specifically, the cellranger count module was applied to each sample for alignment against the Mouse GENCODE reference genome (vM25, released November 2019), cell calling, and UMI counting, thereby generating gene expression matrices (default parameters). No custom modifications were applied to the reference genome.

Data filtering and quality control. Cells with fewer than 200 detected genes and genes expressed in fewer than 3 cells were removed. Cells with a mitochondrial gene content between 5% and 30% (threshold determined by the 99th percentile) and those with >5% hemoglobin gene expression were excluded. Potential doublets were identified and removed via DoubletFinder (v2.0.4).

Normalization, integration, and downstream analysis were performed via SCTransform in Seurat (v4.3.0.1), with the top 2000 highly variable genes selected as anchor features for sample integration. Linear dimensionality reduction was performed via principal component analysis (PCA), and the top 20 principal components were used for downstream analyses. Nonlinear dimensionality reduction for visualization was performed with UMAP and t-SNE. Cell clustering was carried out via Seurat’s FindClusters function with a resolution parameter of 0.6. All subsequent analyses, including normalization, dimensionality reduction, clustering, and annotation on the basis of canonical marker genes, were performed in Seurat. The Single-cell RNA sequencing and analysis were conducted by Chi-Biotech (Shenzhen, China).

### RNA-editing analysis

The adapters of the RNA sequencing reads were removed via Trim Galore (v 0.6.6) with default parameters, and low-quality reads were filtered via fastp (v 0.20.1) with the following parameters: -q 20 -u 20 -n 10 -l 60. The remaining reads were mapped via HISAT2 (v2.2.1) against the human reference genome DNA (build GRCh38.94) with default parameters. REDITools (v2.0)^62^ was applied for the identification of A-to-I editing sites from the BAM files with the following parameters: selectPositions.py (-f 0.1 -e), and AnnotateTable.py (-u -c 1,2,3 -n RepMask). The identified A-to-I RNA-editing sites on target genes were located and visualized via the Integrative Genomics Viewer (IGV).

### Statistical analysis

Statistical analyses were performed via GraphPad Prism (v9.0.0). One-way or two-way ANOVA combined with Tukey’s multiple comparison test was used to calculate P-values. The data are presented as the means ± standard errors of the means (SEMs). P < 0.05 was considered significant. The number of biological (nontechnical) replicates for each experiment is indicated in the figure legends. Statistical comparisons were performed via unpaired t-tests (two-tailed) or Wilcoxon rank-sum tests and log-rank tests, as indicated in the figure legends. The growth of the tumors over time was analyzed via one-way or two-way ANOVA.

## Supplementary information

Supplementary Table 1

Supplementary_Materials

Uncropped Western blots

## Data Availability

The conclusions of this study are supported by data provided in the main text and Supplementary Materials. The transcriptomic datasets generated in this study can be found in the NCBI Sequence Read Archive (SRA) under BioProject accession numbers PRJNA1307764, PRJNA1310172, and PRJNA1310998. The raw MeRIP-seq data are available under PRJNA1310998, the raw whole-transcriptome data are available under PRJNA1310172, and the raw single-cell data are available under PRJNA1307764.
