# Supplementary material for: Exogenous Epstein–Barr virus nuclear antigen 1 induces ADAR1-driven tumor resistance against immunotherapy
Source: Signal Transduct Target Ther. 2026 Feb 18;11:63. doi: 10.1038/s41392-026-02574-y (PMC12913968; doi:10.1038/s41392-026-02574-y)
Supplement: Supplementary file 2 — Supplementary_Materials [file 41392_2026_2574_MOESM2_ESM.docx]

Supplementary Materials for

**Exogenous Epstein-Barr virus nuclear antigen 1 Induces ADAR1-driven** **Tumor Resistance against Immunotherapy**

Changlin Liu^1,4,^ ^#^, Zhiqiang Sun^2,6,^ ^#^, Chao Li^5,^ ^#^, Yanqing Zhou^1,10,^ ^#^, Xuefeng Gao^1,^ ^#^, Yuping Zhong^3^, Xiaomin Luo^1^, Chenci Wang^7^, Yuanbin Zhang^1^, Chuping Ni^1^, Manli Peng^1^, Weiquan Jian^1^, Yinggui Yang^1^, Xuewen Zhang^2^, Yichang Ren^2^, Xinqi Gong^8^, Min Zhao^9^, Xia Guo^1*^, Chao Cheng^3*^, Jianjun Chen^2,6*^, Xin Li^1,10*^

.

These authors contributed equally: Changlin Liu, Zhiqiang Sun, Chao Li, Yanqing Zhou, Xuefeng Gao.

Correspondence to: [lixin@smu.edu.cn](mailto:lixin@smu.edu.cn) (Xin Li); [jchen21@smu.edu.cn](mailto:jchen21@smu.edu.cn) (Jianjun Chen); nurswan@126.com (Chao Cheng); [myshow0504@smu.edu.cn](mailto:myshow0504@smu.edu.cn) (Xia Guo)

**This PDF file includes:**

Materials and Methods

Figures. S1 to S10

Tables S2

**Other Supplementary Materials for this manuscript include the following:**

Uncropped western blots

Materials and Methods

**PBMC- humanized NCG mice**

Four-week-old female NCG mice were housed under SPF conditions in a controlled environment (20–22 °C, 12 h light/12 h dark cycle, 50–70% relative humidity) with free access to food and water. C666-1 cells (1.0×10^7^) were subcutaneously injected into each mouse. Tumor formation was observed after 7 days. Once palpable tumors developed, T cells (5 × 10⁷) presenting tumor antigens derived from C666-1 cell lysates were intravenously transferred into the mice via the tail vein. Subsequently, according to group allocation, mice received intraperitoneal injections of the indicated treatments (DMSO, EP-1215, PD-1 inhibitor, or EP-1215 + PD-1 inhibitor), and tumor tissues were collected at designated time points. Tumor volume was calculated using the formula 0.5 × L × W², where L and W represent the long and short diameters of the tumor, respectively. The laboratory technician responsible for animal care and tumor growth measurements was blinded to group allocation throughout the experiments and outcome assessments.

**In vitro IFNs Treatment and ELISA analysis**

Tumor cells were seeded at 4 × 10⁵ cells per well in 6-well plates and cultured to ~50% confluence. The cells were then treated with IFNβ (100 ng/ml; Sino biological), IFNγ (50 ng/ml; Sino biological), or IFNβ (100 ng/ml) + IFNγ (50 ng/ml) for 6 hours. The cells were washed once with PBS to remove residual interferons, replenished with fresh medium. After a 72-hour of incubation at 37 °C in a 5% CO₂ humidified incubator, the cells supernatant was collected. The concentration of IFNβ or IFNγ were detected using Human Interferon Beta ELISA Kit (Jianglai, JL19215) and Human Interferon Gamma ELISA Kit (Jianglai, JL12152), respectively.

**Cytokine analysis of tumor lysates**

On day 12 post-inoculation, tumors were isolated from mice. 100 mg of tissue was collected into a 2 ml grinding tube containing 500 µl of pre-chilled RIPA buffer (Solarbio, R0010) supplemented with 1 mM PMSF (Roche, 10837091001), complete Protease Inhibitor Cocktail (Roche, 11836170001), and PhosSTOP Phosphatase Inhibitor Cocktail Tablets (Roche, 04906837001). The tissue was homogenized using 2 mm stainless steel beads in a Homogenizer (Servicebio, SWE-3D) at a speed of 6 m/s for 30 seconds, paused for 15 seconds, and cycled 30 times, followed by centrifugation at 16,000g for 15 minutes at 4°C. Protein concentration was assessed using a BCA assay (ThermoFisher, A53225), and the tissue lysates were standardized to 10 mg/ml. The levels of IFNβ or IFNγ protein in the lysates were then measured using Mouse Interferon Beta ELISA Kit (Jianglai, JL20219) and Mouse Interferon Gamma ELISA Kit (Jianglai, JL10967).

**In vitro cell viability assay**

Cell viability was measured using the CellTiter-Lumi™ Plus II kit (Beyotime, C0065S) based on ATP content. Tumor cells were initially seeded at a density of 1 × 10⁴ cells per well in a 96-well plate containing the following cytokine combinations: IFNβ (100 ng/ml; Sino Biological), IFNγ (50 ng/ml; Sino Biological), or IFNβ (100 ng/ml) + IFNγ (50 ng/ml). After 72-hour incubation, cells were lysed, and CellTiter-Lumi™ reagent was added according to the manufacturer’s instructions. Six replicates were established for each condition, and chemiluminescence intensity was subsequently recorded using a multifunctional microplate reader. The average values were used to calculate cell viability.

**Live-cell imaging co-culture assay**

GFP-labeled tumor cells were seeded at a density of 2,000 cells per well in 96-well plates with 100 µl of medium and allowed to reach approximately 30% confluence. After 6 days of Primary T cells activation, Primary T cells were added to tumor cells at various concentrations for co-culture, with the final volume adjusted to 200 µl per well. The plates were equilibrated at room temperature for 30 minutes, then transferred to live-cell imaging analysis system for continuous live-cell imaging. Tumor cell viability was monitored every 1-hour images using live-cell analysis system (Sartorius, IncuCyteSx3), based on changes in fluorescence area to evaluate T cell-mediated cytotoxicity. Survival of tumor cells was quantified using the proprietary analysis software (Sartorius, IncuCyte).

**Immunohistochemistry**

Tissue sections were fixed in 4% paraformaldehyde for 24-48 hours, dehydrated through graded ethanol solutions, and embedded in paraffin. Sections (3-7 µm thick) were deparaffinized in xylene, rehydrated through graded ethanol, and subjected to antigen retrieval in Tris-EDTA buffer (pH 9.0) at 95°C for 10 minutes. Endogenous peroxidase activity was blocked with 3% H₂O₂ for 10 minutes, followed by blocking with 2% BSA for 30 minutes at room temperature. Sections were then incubated with primary antibody overnight at 4°C, followed by incubation with HRP-conjugated secondary antibody for 1 hour at 37°C. The DAB substrate was used for color development, and slides were counterstained with hematoxylin. After dehydration and mounting, images were captured under a microscope.

**Tissue array Construction**

Tissue arrays were constructed by selecting representative regions from paraffin-embedded tissue blocks. Cores with a diameter of 2 mm were punched from donor blocks and precisely arrayed into recipient blocks, with each core spaced 0.2 mm apart. The assembled TMAs were sectioned at 4 µm thickness, mounted onto slides, and stored at -20°C until further analysis.

**Immunofluorescence**

Cells were seeded on glass coverslips in 24-well plates at a density of 1 × 10⁴ cells per well and allowed to adhere overnight. After treatment, cells were fixed with 4% paraformaldehyde for 15 minutes at room temperature, permeabilized with 0.2% Triton X-100 for 5 minutes, and blocked with 3% BSA for 30 minutes. Cells were incubated overnight at 4°C with primary antibodies, including anti-EBNA1 (Proteintech, 21921-1-AP) and anti-IGF2BP3 (Abclonal, A15199). After washing three times with PBS, Alexa Fluor-conjugated secondary antibodies (e.g., Alexa Fluor 488 goat anti-rabbit IgG, Thermo Fisher, A-11008 and Alexa Fluor 594 goat anti-mouse IgG, Thermo Fisher, A-11005) were added and incubated for 1 hour at room temperature in the dark. Nuclei were counterstained with DAPI. Coverslips were mounted onto slides with anti-fade mounting medium, and fluorescence images were captured using a fluorescence microscope.

**Co-Immunoprecipitation and LC-MS/MS analysis**

Cells were cultured in 15 cm dishes until they reached 90% confluence, and then lysed in IP lysis buffer (ThermoFisher, 87787) supplemented with protease and phosphatase inhibitors. After centrifugation at 12,000 × g for 15 minutes at 4°C, the supernatant was collected, and protein concentration was measured using a BCA assay kit. Equal amounts of protein lysates were incubated with 1 μg of primary antibody on a rotator at room temperature for 2 hours, followed by the addition of 20 µl of Protein A/G MagPoly Beads (Smart-Lifesciences, SM015010), and incubated overnight on a rotator at 4°C. The beads and immune complexes were then washed five times with precooled IP lysis buffer. LC-MS/MS analysis (Liquid Chromatography–Tandem Mass Spectrometry) was conducted by chi-biotech. Separation was performed using a nano-HPLC system (Easy-NLC1200). The hydrolysates were separated by capillary high-performance liquid chromatography and analyzed using a Q-Exactive mass spectrometer. MS/MS spectra were searched with MaxQuant (v2.0.3.0) against the UniProt Homo sapiens database. After adding a certain volume of SDS loading buffer (ThermoFisher, LC2676), the samples were boiled at 95°C for 10 minutes. Protein interactions were confirmed by Western blotting to detect the corresponding proteins.

**RNA extraction, reverse transcription and RT-qPCR**

Total RNA was extracted using the Trizol reagent (ThermoFisher, 15596018CN) according to the manufacturer’s protocol. Briefly, cells were homogenized in Trizol, incubated at room temperature for 5 minutes, and then chloroform was added. After vigorous shaking and centrifugation at 12,000 × g for 15 minutes at 4°C, the aqueous phase containing RNA was transferred to a new tube. RNA was precipitated by adding isopropanol and centrifuged at 12,000 × g for 10 minutes at 4°C. The RNA pellet was washed with 75% ethanol, air-dried, and dissolved in DEPC-treated water. RNA concentration and purity were determined using a Nanodrop spectrophotometer, and samples were stored at -80°C.

The extracted RNA was reverse transcribed into cDNA using the All-in-One First-Strand cDNA Synthesis Kit (TransGen, AW311-03) according to the manufacturer's instructions.

The real-time qPCR mixture was prepared according to the manufacturer's instructions using the PerfectStart Green qPCR SuperMix kit (supplemented with Dye II; Transgen, AQ132-21), and the reaction was run on an ABI Prism 7500 (ABI). The expression level of each mRNA was normalized to the mRNA level of the housekeeping gene GAPDH, Relative gene expression was calculated using the 2^–ΔΔCT^ method. The following is a list of primers used in RT-qPCR (5ʹ to 3ʹ):

ADAR1-F (Full-length): CTGAGACCAAAGAAAGCAGCAG

ADAR1-R (Full-length): GCCACTTGTAGATGAGGACGGT

ADAR1-F (RIP and MeRIP): TTCAGGGCAGAGGAGTCAGA

ADAR1-R (RIP and MeRIP): AAATCGCGGTCTCCACTCAG

GAPDH-F: GGAGCGAGATCCCTCCAAAAT

GAPDH-R: GGCTGTTGTCATACTTCTCATGG.

**RNA pull-down assays**

Biotin-labeled RNA oligonucleotides containing A or m^6^A were synthesized by GeneCreate (China). Each RNA oligonucleotide (0.4 pmol, unless otherwise specified) was immobilized onto 50 μL of streptavidin magnetic beads (Thermo Fisher Scientific). The RNA pull-down assay was performed using the Magnetic RNA Protein Pull-Down Kit (Thermo Fisher, 20164) according to the manufacturer’s instructions. Briefly, RNA oligonucleotides containing A, m^6^A (biotin-labeled) or ADAR1 mRNA (synthesized by in vitro transcription and biotin-labeled) were immobilized onto streptavidin magnetic beads and incubated with binding buffer (20 mM Tris, 200 mM NaCl, 6 mM EDTA, 5 mM potassium fluoride, 5 mM β-glycerophosphate, 2 μg/mL aprotinin, pH 7.5) at 4°C for 4 hours. The RNA bait-conjugated streptavidin beads were then incubated overnight with tumor cell lysates in binding buffer at 4°C on a rotator. After extensive washing, the RNA-protein-bead complexes were dissolved in 1× SDS buffer, separated under denaturing conditions on 10% SDS-polyacrylamide Bis-Tris gels, and detected by western blot analysis. For mass spectrometry analysis, the RNA-protein-bead complexes were sent to Chi-Biotech for further detection and analysis.

**RNA stability assay**

Cells were seeded in 12-well plates at approximately 60% confluency and allowed to adhere overnight. Actinomycin D was added to the culture medium at a final concentration of 2 µg/ml. Cells were harvested at 0, 20,40, 60,and 80 minutes post-treatment. Total RNA was extracted from each time point using the Trizol method, followed by reverse transcription. Gene expression levels were subsequently analyzed by RT-qPCR.

**Dual-Luciferase Reporter Assay**

The ADAR1 wild-type (WT) and mutant (Mut) reporter constructs were designed based on MeRIP-Seq results to evaluate the binding affinity of target sequences. Constructs were generated by inserting WT or Mut sequences into a dual-luciferase reporter plasmid. HK1 cells were co-transfected with WT or Mut reporter constructs along with EBNA1 or control plasmids using Lipofectamine 3000 (Thermo Fisher, L3000015). After 48 hours, cells were lysed in 20 µl of passive lysis buffer, and 10 µl of lysate was transferred to a 96-well plate for analysis. The dual-luciferase assay was performed by sequentially adding 100 µl of firefly luciferase reagent followed by Renilla luciferase reagent according to the manufacturer's protocol (Yeasen Biotechnology, 11402ES80). Luminescence was measured using a microplate reader. Relative luciferase activity was calculated as the ratio of firefly (F-Luc) to Renilla (R-Luc) luminescence.

**Primary T Cells Isolation and Culture**

Peripheral Blood Mononuclear Cells (PBMCs) were isolated from the fresh blood of healthy donors using LymphoprepTM (STEMCELL, 18061) density gradient centrifugation. Isolated PBMCs were washed with PBS and resuspended in RPMI-1640 medium with 10% FBS. The isolated PBMCs were washed three times with PBS containing 2% FBS, and then resuspended in RPMI-1640 medium with 10% FBS for culture.

To activate and expand T cells, IL-2 (100ng/ml; ThermoFisher, 200-02-50UG) and DynabeadsTM Human T-Expander CD3/CD28 (Gibco, 11141D) were added to the medium containing PBMCs, and cultured at 37°C in a 5% CO₂ incubator. The bead-to-cell ratio is 2:1. The medium was refreshed every 2-3 days.

**Lentivirus, plasmids and transfection**

The pCDH-CMV-GFP-Puro vector was utilized to overexpress EBNA1 with a 3xFlag tag. The EBNA1 coding sequence was amplified using specific primers (forward: CTCCATAGAAGATTCTAGAGCTAGCG, reverse: AATCACCCTGATGTCTTTGTAGTCC) and cloned into the BamHI and EcoRI sites of the vector. The ligated plasmid was transformed into DH5α cells, and positive clones were confirmed by colony PCR and sequencing. HEK293FT cells were co-transfected with pCDH-EBNA1-GFP-Puro and the packaging plasmids pSPAX2 and pMD2.G using Lipofectamine 3000. After 48 hours, viral supernatants were collected, filtered, and concentrated by ultracentrifugation. The viral particles were resuspended in PBS and stored at -80°C. Target cells were infected with lentiviral particles in the presence of polybrene (10 µg/ml). After 24 hours, the medium was replaced, and cells were cultured for an additional 48 hours. Transduced cells were selected with puromycin (1-10 µg/ml) for 3-5 days. Western blot was used to confirm transduction efficiency and EBNA1 expression. The shRNA plasmid targeting human IGF2BP3 (based on the pcDNA3 vector) was kindly provided by Professor Hui-Lin Huang from the Sun Yat-sen University Cancer Center. The target sequences 5'-GATCGGAAGGAAGGTGAGAA-3' or 5'- GATCGGTGTCCAAGCAGAAA-3' were inserted into the pLV3-U6-Fluc-puro vector to construct two plasmids targeting IGF2BP3. The sequences 5'- GATCGGTGTCCAAGCAGAAA -3' or 5'- AGATATAAATGCTGTGCTAAT -3' were inserted into the pLV3-U6-Fluc-puro vector to construct two plasmids targeting human ADAR1. To construct two plasmids targeting human EIF4G1, the following sequences were inserted into the pLKO.1-u6-puro vector: 5'- GTCATCCCGCATCCGCTTTAT -3' and 5'- GTCTGCTATTCTGCAATTATT -3'. The construction of IGF2BP3-HA MUT1, MUT2, MUT3, MUT4, and GxxGΔ (GxxG mutated to GEEG) mutants was performed as previously described^1^.

**Flow Cytometry (FCM)**

Tumors were collected, finely minced, and digested with an enzyme mixture in 2% FBS 1640 medium at 37°C for 30 minutes. The resulting cell suspension was filtered through a 70 µm strainer, centrifuged at 500 g for 5 minutes, washed with PBS, and then incubated with FcR blocking antibody (Biolegend, 101320) for 30 minutes. Cells were then stained with the appropriate antibodies in the dark at 4°C for 30 minutes. For intracellular staining, cells were first fixed in fixation buffer (Biolegend, 420801), followed by staining with the target antibody in permeabilization buffer (Biolegend, 421002). Flow cytometry analysis was performed on a SA3800 cytometer (SONY), and the data were processed using the cytometer’s built-in software (SONY).

**Polysome profiling analysis**

The cells to be extracted were pre-treated with 100 μg/ml cycloheximide (CHX;) MCE, HY-12320) and incubated at 37°C for 15 minutes. They were then washed three times with ice-cold PBS containing 150 μg/ml CHX and collected. Lysis buffer was added, and after cell disruption, intermittent shaking was performed using a low-temperature shaker. The lysis step was carried out on ice. After lysis, the samples were centrifuged, and the supernatant was collected. The supernatant was gently dripped onto a pre-prepared 5-50% linear sucrose gradient and equilibrated. The samples were then ultracentrifuged at 36,000 rpm for 2 hours at 4°C. The samples were subsequently fractionated and analyzed using a Piston Gradient Fractionator (Biocomp) and fraction collector (Gilson). 5 ng of polyadenylated synthetic luciferase mRNA (Promega) was added to each fraction for normalization. RNA was extracted from each fraction and subjected to qRT-PCR analysis.

**Western Blotting**

Cell lysates were collected in RIPA buffer (Thermo Fisher, 89900), with protease inhibitor tablets (Roche, 11836170001) and phosphatase inhibitor tablets (Roche, 04906837001) added according to the manufacturer's instructions. For the detection of ADAR1 downstream sensor molecules (MDA5, MAVS, RIG-I, PKR, p-PKR), proteins were collected following stimulation with IFNβ (100 ng/ml) or Poly I:C (2 ug/ml; MCE,42424-50-0). Total protein was obtained by centrifugation, and protein concentration was determined using the Pierce BCA Protein Assay Kit (Thermo Fisher, A53225). Equal amounts of total protein were separated by 8–12% SDS-PAGE (Epizyme Biotech) and transferred onto a 0.45 µm NC membrane (Millipore, HATF00010). The membrane was immunoblotted with the indicated primary antibody and then incubated with HRP-conjugated secondary antibody. Blots were incubated with ECL substrate (BioRad, 1705061) and imaged with the ECL detection system (BioRad, ChemiDoc).

**RIP**

Cells were harvested at 90% confluence, washed with PBS, and lysed in lysis buffer supplemented with RNase and DNase inhibitors. The lysate was incubated with Protein A/G magnetic beads pre-bound to 2 µg of primary antibody and gently rotated at 4°C overnight. An IgG antibody was used as a control. Beads were washed three times with RIP buffer (150 mM KCl, 25 mM Tris [pH 7.4], 5 mM EDTA, 0.5 mM DTT, 0.5% NP40, 1× protease inhibitor) to remove unbound material. RNA-protein complexes were then eluted and treated with Proteinase K to digest protein. RNA was extracted from the complexes using Trizol reagent and purified. The concentration and quality of RNA were determined using a Nanodrop spectrophotometer. The extracted RNA was subsequently used for RT-qPCR or RIP-seq analysis.

**MeRIP**

m^6^A RNA immunoprecipitation was performed according to the instructions of the Magna MeRIP m^6^A kit (Merck, 17-10499). Total RNA was first extracted using the Trizol method and fragmented into ~100 nt pieces. m^6^A-seq library preparation and sequencing were carried out by OE Biotech Co., Ltd (Shanghai, China). The fragmented RNA was divided into two portions. One portion was incubated with pre-mixed m^6^A antibody–conjugated magnetic beads to enrich m^6^A-methylated mRNA fragments. The other portion served as a control and was directly used for conventional transcriptome library construction. After enrichment, the m^6^A-containing mRNA fragments were recovered and subjected to standard transcriptome library preparation. The two constructed sequencing libraries, namely the m6A-seq library and the RNA-seq library, were then subjected to high-throughput sequencing on the Illumina NovaSeq 6000 platform using the PE150 sequencing mode. The raw reads generated from high-throughput sequencing were quality-controlled with fastp to obtain high-quality clean reads. Clean reads were aligned to the human reference genome (GRCh38/hg38, Ensembl release 109) using Hisat2 with default parameters. The alignment results were further assessed with the Guitar R package and deeptools software to evaluate data quality and verify the efficiency of the RIP-seq experiment.

**Isolation of CD45^+^ Immune Cells**

Tumor tissues were processed through a 30% Percoll gradient and centrifuged at 600 g for 20 minutes to remove debris. The cell suspension was then washed with PBS, resuspended in MACS buffer, and treated with Dead Cell Removal MicroBeads (Miltenyi Biotec) according to the manufacturer's protocol to exclude non-viable cells. For CD45^+^ cell isolation, viable cells were incubated with CD45 MicroBeads (Miltenyi Biotec) and then passed through a MACS column (Miltenyi Biotec) under a magnetic field. The enriched CD45^+^ cells were resuspended in MACS buffer, counted, and assessed for purity and viability.

**Antibodies**

Flow cytometry antibodies are listed in Supplementary Table 2. Except for the immunofluorescence primary antibody IGF2BP3 polyclonal antibody (Proteintech, CL488-66526) and the immunohistochemistry antibody CD8A (Abclonal, A22219), the remaining antibodies used in immunofluorescence and immunohistochemistry experiments are the same as those used in western blotting experiments, including IGF2BP1 monoclonal antibody (Abclonal, A22612), IGF2BP2 monoclonal antibody (Abclonal, A5189), IGF2BP3 polyclonal antibody (Proteintech, 14642-1-AP), ADAR1 monoclonal antibody (Abclonal, A11466), EIF4G1 polyclonal antibody (Proteintech, 15704-1-AP), MDA5 polyclonal antibody (Proteintech, 21775-1-AP), MAVS antibody (Proteintech, 81910-1-RR), PKR polyclonal antibody (Proteintech, 18244-1-AP), RIG-I/DDX58 polyclonal antibody (Proteintech, 20566-1-AP), Phospho-PKR (Thr446/451) antibody (CST, 3071), FLAG tag antibody (Sigma, F1804), HA tag polyclonal antibody (Proteintech, 51064-2-AP), GAPDH (YEASEN, 30201ES60), EBNA1 monoclonal antibody (Santa Cruz, sc-81584), HRP-conjugated Goat anti-Rabbit IgG (H+L) (Abclonal, AS014), HRP-conjugated Goat anti-Mouse IgG (H+L) (Abclonal, AS003). For IP experiments, the control antibody used is Rabbit IgG control polyclonal antibody. Immunofluorescence secondary antibodies are as follows: Alexa Fluor^™^568 conjugated Goat anti-Mouse IgG H+L (Thermo Fisher, A-11031), Alexa Fluor^™^647 conjugated Goat anti-Rabbit IgG H+L (Thermo Fisher, A-21245), Alexa Fluor^™^647 conjugated Donkey anti-Mouse IgG H+L (Thermo Fisher, A-31571), Alexa Fluor^™^568 conjugated Goat anti-Rabbit IgG H+L (Thermo Fisher, A-11036).

**References:**

1 Huang, H. *et al.* Recognition of RNA N(6)-methyladenosine by IGF2BP proteins enhances mRNA stability and translation. *Nat Cell Biol* **20**, 285-295.

**Supplementary Figures**


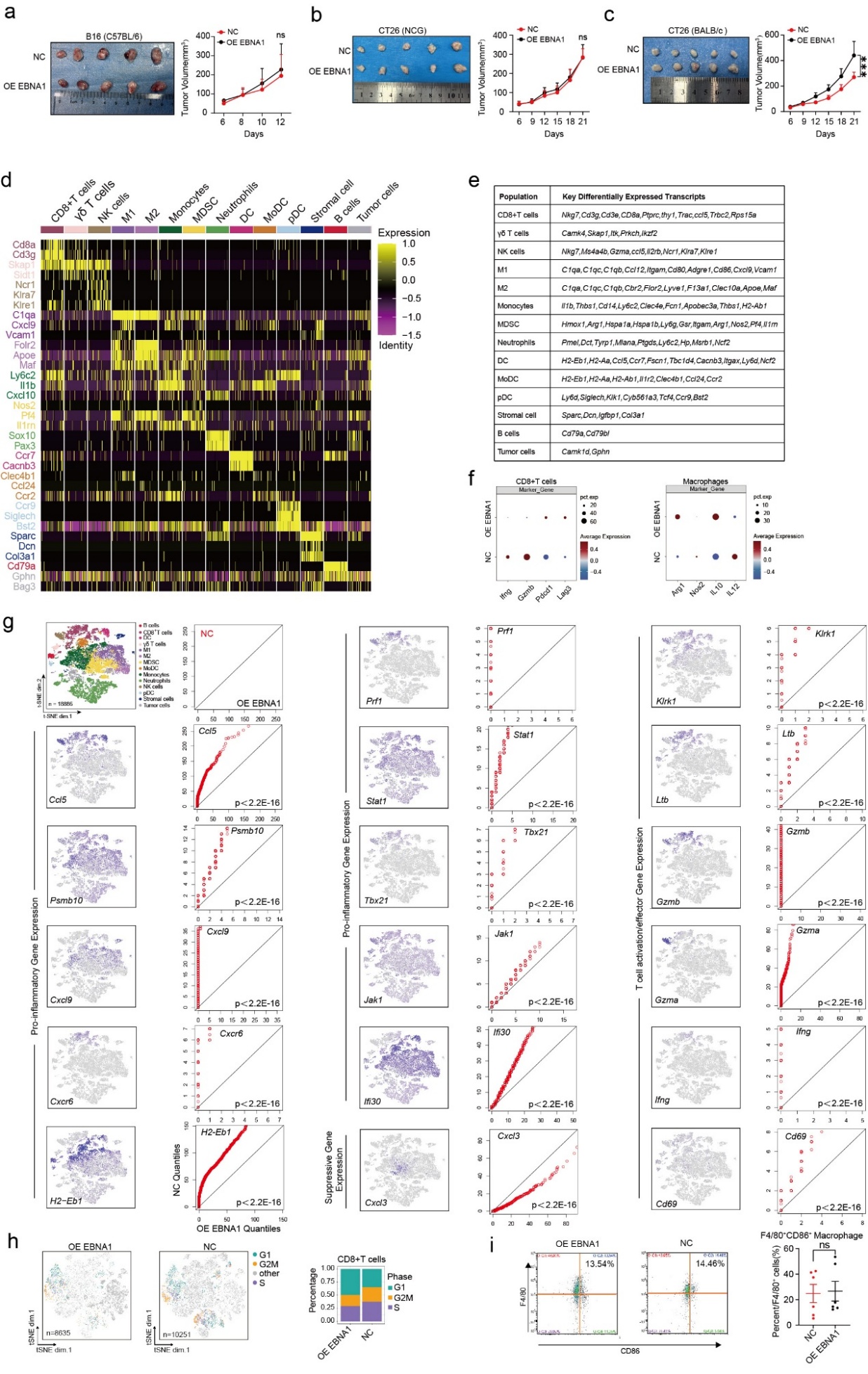


**Supplementary Figure 1.** **Single-cell RNA sequencing extended data and FACS validation.a**, Representative images of tumors from NCG mice injected with either NC or OE EBNA1 B16 cells (left), along with the corresponding tumor volume (right). (n=5 mice in each group). **b**, Representative images of tumors from NCG mice injected with either NC or OE EBNA1 CT26 cells (left), along with the corresponding tumor volume (right). (n=5 mice in each group). **c**, Representative images of tumors from BALB/c mice injected with either NC or OE EBNA1 CT26 cells (left), along with the corresponding tumor volume (right). (n=5 mice in each group). **d**, Gene expression matrix from a single-cell RNA-seq experiment characterizing the expression of lineage-defining genes across different cell clusters. **e**, Key differentially expressed transcripts distinguishing the cell clusters shown in **Fig. 1. f**, Expression levels of key molecules in CD8⁺ T cells (IFNG, Gzmb, Pdcd1, Lag3) and macrophages (Arg1, Nos2, IL10, IL12) within NC or OE EBNA1 tumors. **g**, Paired quantile-quantile (Q-Q) plots were used to compare the expression of selected genes in immune cells from OE EBNA1 and NC tumors, alongside matched t-SNE plots illustrating the gene expression distribution for proinflammatory, suppressive, and T cell activation/effector genes. P values were determined using the Wilcoxon rank-sum test. **h**, Comparison of the cell cycle of immune CD8^+^T cells in EBNA1 overexpressing tumors and control tumors. **i**, Representative FACS images (left) and statistical analysis (right) of M1 macrophages in OE EBNA1 tumors and NC tumors. There are 6 samples per group(n=6). Two-tailed unpaired t-test was used for analysis. ns, no significance.

**a, b, c,** Two-way ANOVA with Tukey’s test for multiple comparisons.


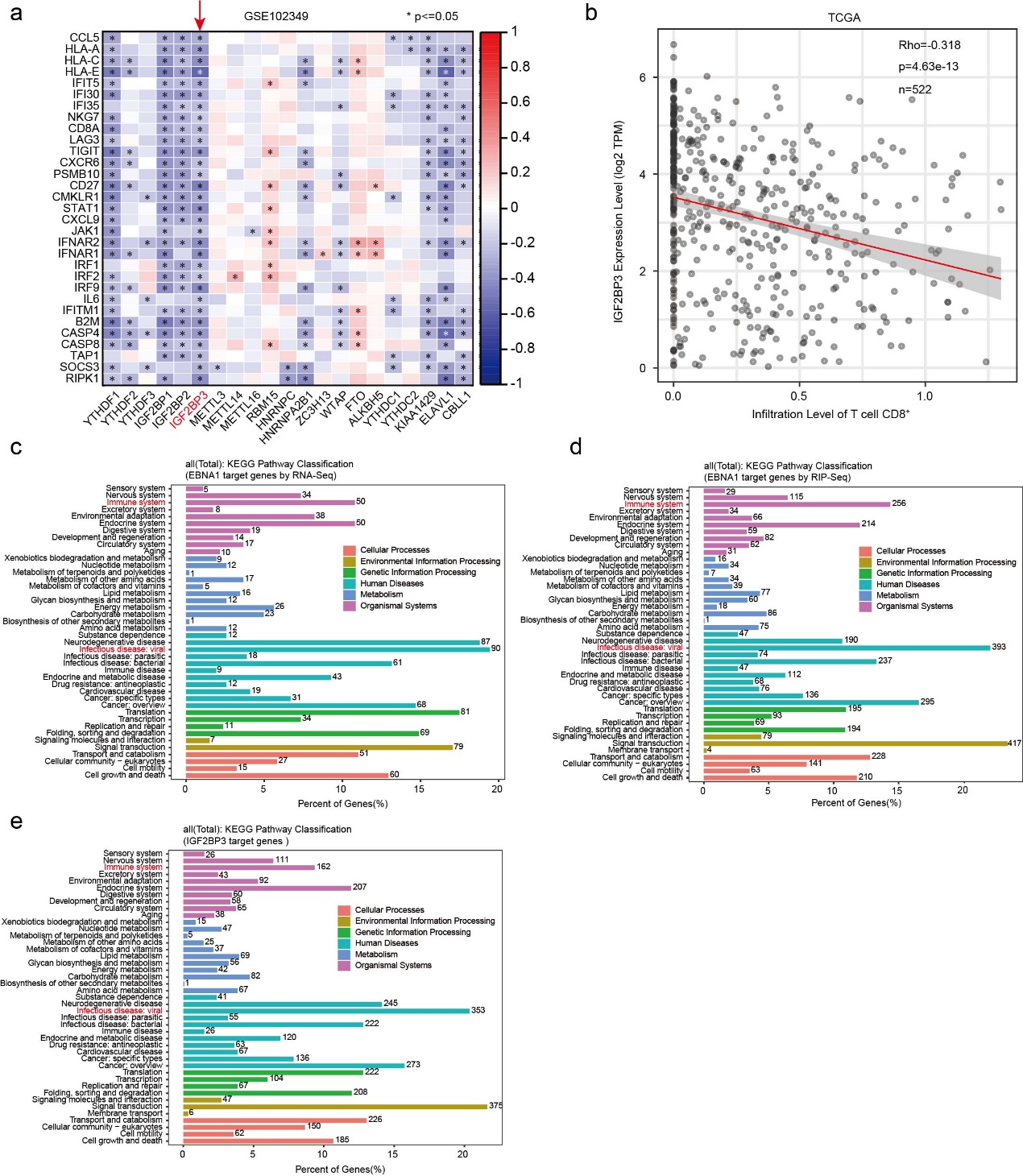


**Supplementary Figure 2. EBNA1 and IGF2BP3 influence the immune response. a**, The Spearman correlation of mRNA expression between interferon-related genes and m^6^A regulatory factors in the GSE102349 dataset. **b**, Spearman's correlation between CD8^+^T cells infiltration and IGF2BP3 expression levels in 522 Head and Neck Squamous Cell Carcinoma (HNSC) cases from TCGA. **c**, KEGG analysis of target genes with increased expression levels following overexpressing EBNA1. **d**, KEGG analysis of EBNA1 target genes identified by RIP-Seq in HK1-EBV cells. **e**, KEGG analysis of IGF2BP3 target genes identified by Huang et al^1^, using RIP-Seq and CLIP-Seq.


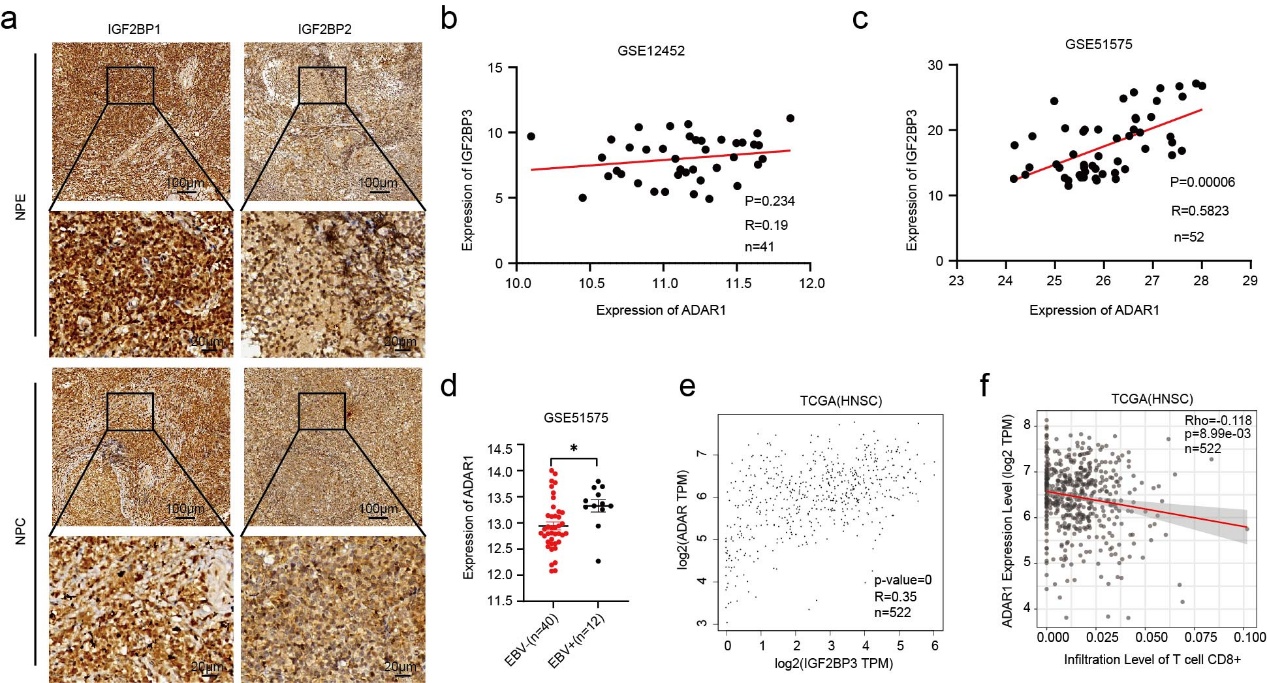


**Supplementary Figure 3. Correlation analysis between ADAR1 and IGF2BP3. a**. Representative IHC images of IGF2BP1 and IGF2BP2 in NPE (n=45) and NPC (n=40) samples are shown (scale bar, 100 µm or 20 µm; magnifications, 100× and 400×). **b**. Pearson's correlation between ADAR1 and IGF2BP3 expression levels in 41 clinical samples (31 NPC samples and 10 NPE samples) from GSE10452. **c**. Pearson's correlation between ADAR1 and IGF2BP3 expression levels in 52 clinical samples (26 gastric tumor and 26 gastric normal) from GSE51575. **d**, ADAR1 expression levels in EBV^-^(n=40) and EBV^+^(n=12) groups were evaluated in the GSE51575 dataset. Data are presented as mean±s.e.m. Two-tailed unpaired t-test was used. *P < 0.05. **e**. Pearson's correlation between ADAR1 and IGF2BP3 expression levels in 522 HNSC cases from TCGA. **f**. Spearman's correlation between CD8^+^T cells infiltration and ADAR1 expression levels in 522 HNSC cases from TCGA.


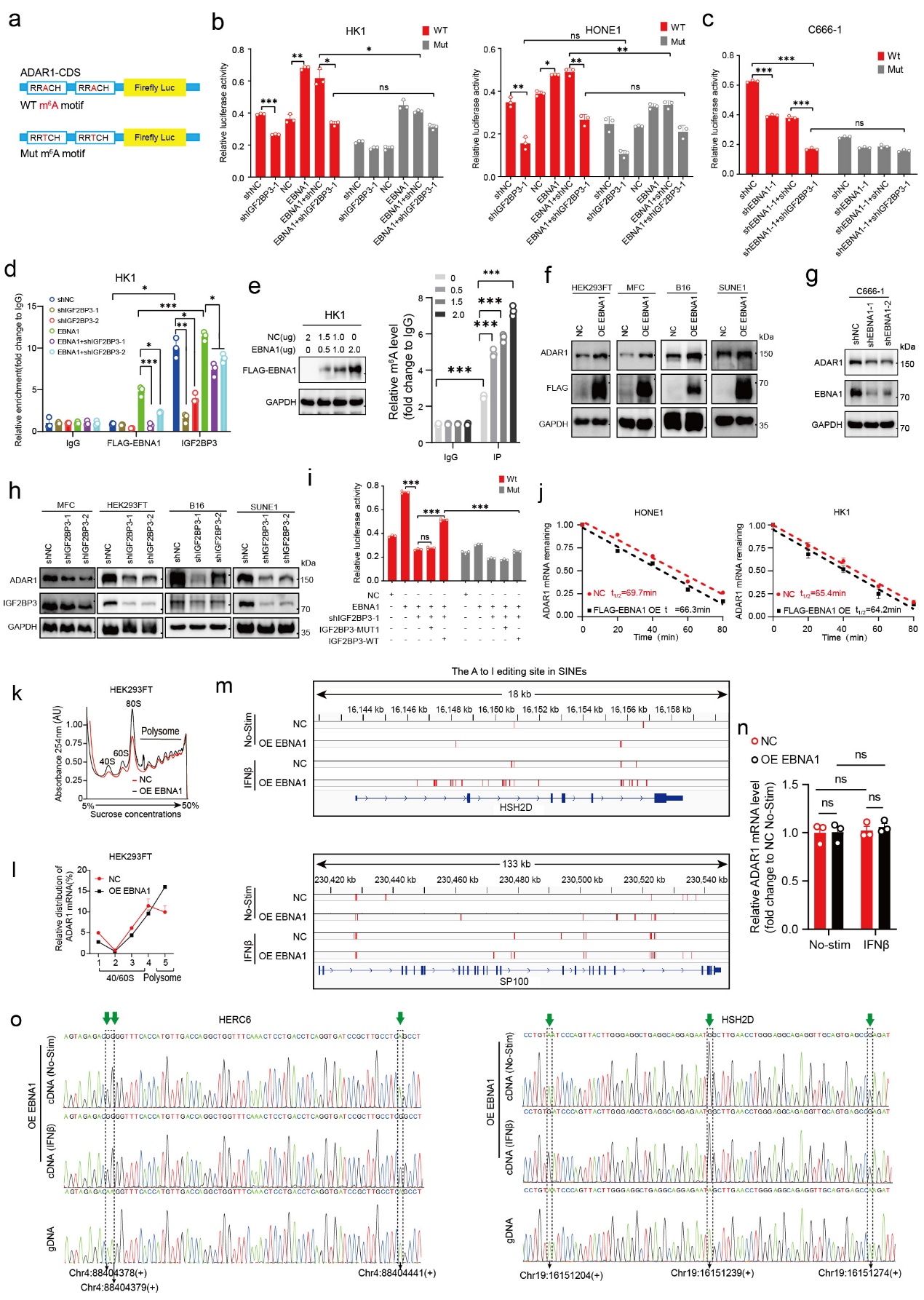


**Supplementary Figure 4. EBNA1 cooperates with IGF2BP3 to regulate ADAR1 through m^6^A modification. a**, A schematic diagram illustrating the fusion of wild-type and mutant m^6^A sites in the ADAR1 coding sequence (CDS) region with a dual-luciferase reporter gene. **b**, ADAR1-CDS wild-type plasmid and ADAR1-CDS m^6^A mutant plasmid were transfected into six groups as shown for 72 hours, and the firefly luciferase activity was measured and normalized to Renilla luciferase activity. **c**, ADAR1-CDS wild-type plasmid and ADAR1-CDS m^6^A mutant plasmid were transfected into four groups (shNC, shEBNA1-1, shEBNA1-1+shNC, shEBNA1-1+shIGF2BP3-1), cultured for 72 hours, and firefly luciferase activity was measured and normalized to Renilla luciferase activity.

**d**, FLAG-RIP-qPCR and IGF2BP3-RIP-qPCR experiments were performed on six cell groups as indicated. **e**, Different amounts of EBNA1 plasmid (0, 0.5, 1.0, 1.5, and 2.0 µg) were transfected into HK1 cells, and the ability of the m^6^A antibody to enrich ADAR1 mRNA was examined after 48 hours. **f**, Following EBNA1 overexpression, western blot showed ADAR1 protein levels in gastric cancer cells (MFC), NPC cells (SUNE1), melanoma cells (B16), and epithelial cells (HEK293FT). **g**, In C666-1 cells, EBNA1 was knocked down to examine changes in ADAR1 protein levels. **h**, After knocking down IGF2BP3 in gastric cancer cells (MFC), NPC cells (SUNE1), melanoma cells (B16), and epithelial cells (HEK293FT), ADAR1 protein levels were detected. **i**, In EBNA1-overexpressing HEK293FT cells, cell lines were generated with IGF2BP3 knockdown followed by reintroduction of IGF2BP3-MUT1 or IGF2BP3-WT. ADAR1-CDS wild-type plasmid and ADAR1-CDS m^6^A mutant plasmid were then transfected into these cells, cultured for 72 hours, and firefly luciferase activity was measured and normalized to Renilla luciferase activity. **j**, After EBNA1 overexpression in HK1 or HONE1 cells, the half-life of ADAR1 mRNA was analyzed by qPCR after treated with Actinomycin D. Data are presented as mean±s.e.m. from three independent experiments. **k**, Sucrose gradient-based polysome profiling of control and EBNA1 overexpressing HEK293FT cells. **l**, ADAR1 mRNA in each polysome fraction was quantified using qRT-PCR and results were plotted as a percentage of the total amount. **m**, In whole transcriptome sequencing, A-to-I editing in the SINEs regions was analyzed and mapped to specific transcript locations (red lines). **n**, In EBNA1-expressing cells induced by IFN and their control groups, qPCR was performed to examine changes in ADAR1 mRNA levels.

**o**, Sanger sequencing validation of genomic DNA (gDNA, bottom) and complementary DNA (cDNA, top and middle) from EBNA1-overexpressing cells with and without IFNβ stimulation. Green arrows indicate adenosine sites that underwent A-to-I editing.

**b**-**e**, **i**, **n**, Data are presented as mean±s.e.m. Two-way ANOVA with Tukey’s test for multiple comparisons. (n = 3 independent experiments). *P < 0.05; **P < 0.01; ***P < 0.001; ns, no significance. **b**-**j**, **n**, The results are representative of three independent experiments.


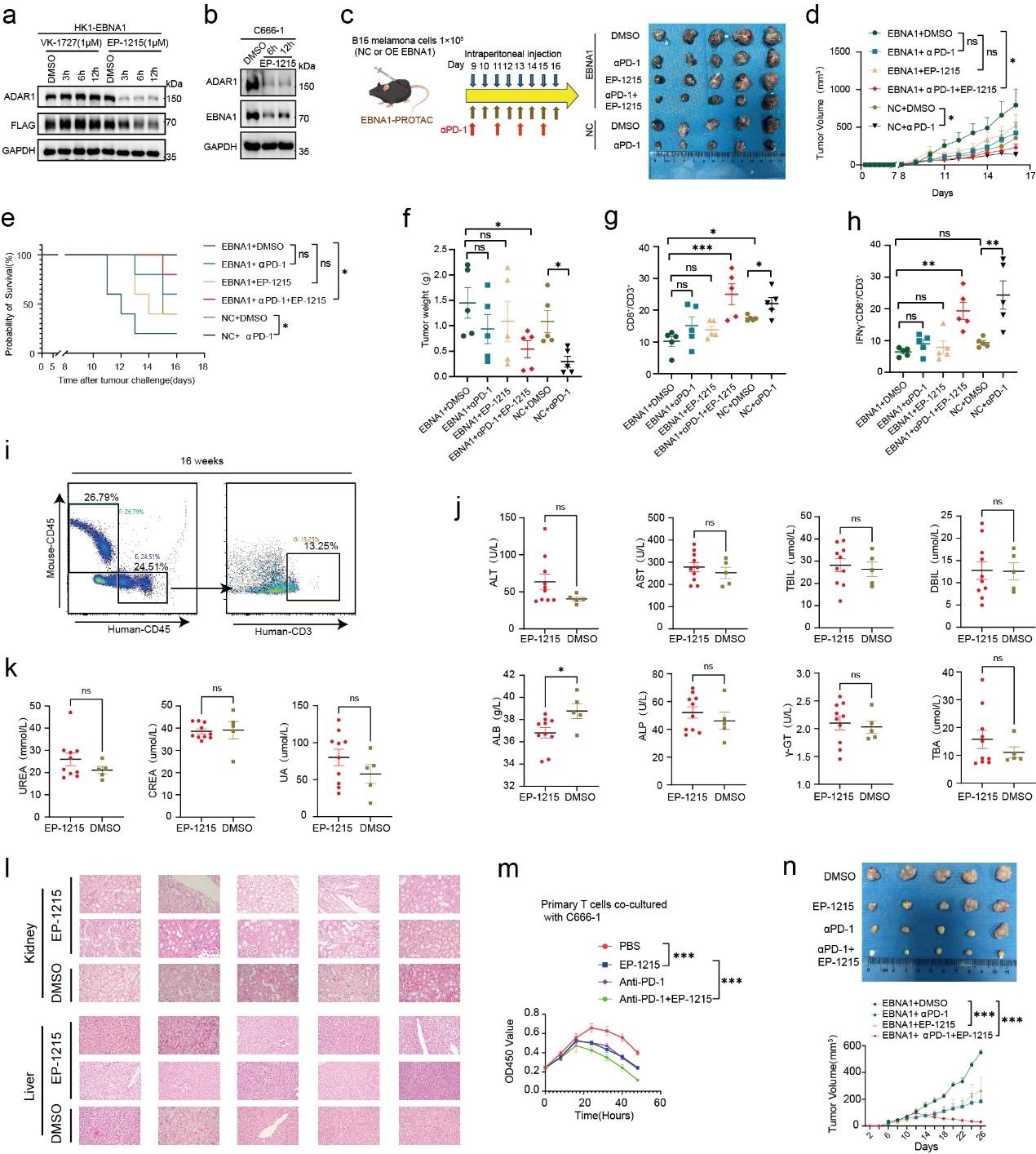


**Supplementary Figure 5. EP-1215 exerts its anti-tumor effects by degrading EBNA1， which reduces ADAR1 protein levels. a**, ADAR1 protein levels were detected after treatment with EBNA1-degrader EP-1215 or EBNA1-inhibitor VK-1727 at various time points.

**b**, In C666-1 cells, EBNA1 and ADAR1 protein levels were examined after treatment with EP-1215. **c**, Xenograft tumors were established by implanting B16 cells (NC or OE EBNA1) into C57BL/6 mouse models, which were treated with DMSO, αPD-1, EP-1215, or a combination of αPD-1 and EP-1215. The mouse diagram was created by Figdraw. Representative images of xenograft tumors for the different groups are shown (right).

**d**, **f**, Tumor volume and tumor weight of the mice shown in **Supplementary Figure 5c** (n = 5 mice per group). **e**, Survival analysis of the mice shown in **Supplementary Figure 5c** (n = 5 mice per group). **g**, **h**, Flow cytometry analyses of CD3^+^CD8^+^T cells and IFNγ^+^CD8^+^T cells in xenograft tumors from C57BL/6 mice. **i**, The percentage of CD45^+^cells were identified from CD34^+^humanized mice using flow cytometry. **j**, **k**, Liver (**j**) and renal (**k**) function indicators were examined in plasma extracted from CD34^+^ humanized mice, which treated with EP-1215 (n=10) or DMSO (n=5). **l**, HE staining was performed to assess the extent of liver and kidney function damage in mice. **m**, C666-1 cells were co-cultured with T cells to evaluate the therapeutic effects of EP-1215, αPD-1 monotherapy, or a combination of EP-1215 and αPD-1 treatment. **n**, By establishing PBMC-humanized mice, we subcutaneously xenografted C666-1 cells and set up four treatment groups: PBS, EP-1215, PD-1, and EP-1215+PD-1. Tumor volumes were measured and growth curves were plotted. **c-h**, **n**, n=5. **d, f**, **g**, **h**, **j**, **k**, **m**, **n**, Data are shown as mean±s.e.m. **d, f**, **g**, **h**, **m**, **n**, Two-way ANOVA with Tukey’s test for multiple comparisons. **e**, log-rank test was used. *P < 0.05;**P < 0.01 ; ***P < 0.001；ns, no significance.


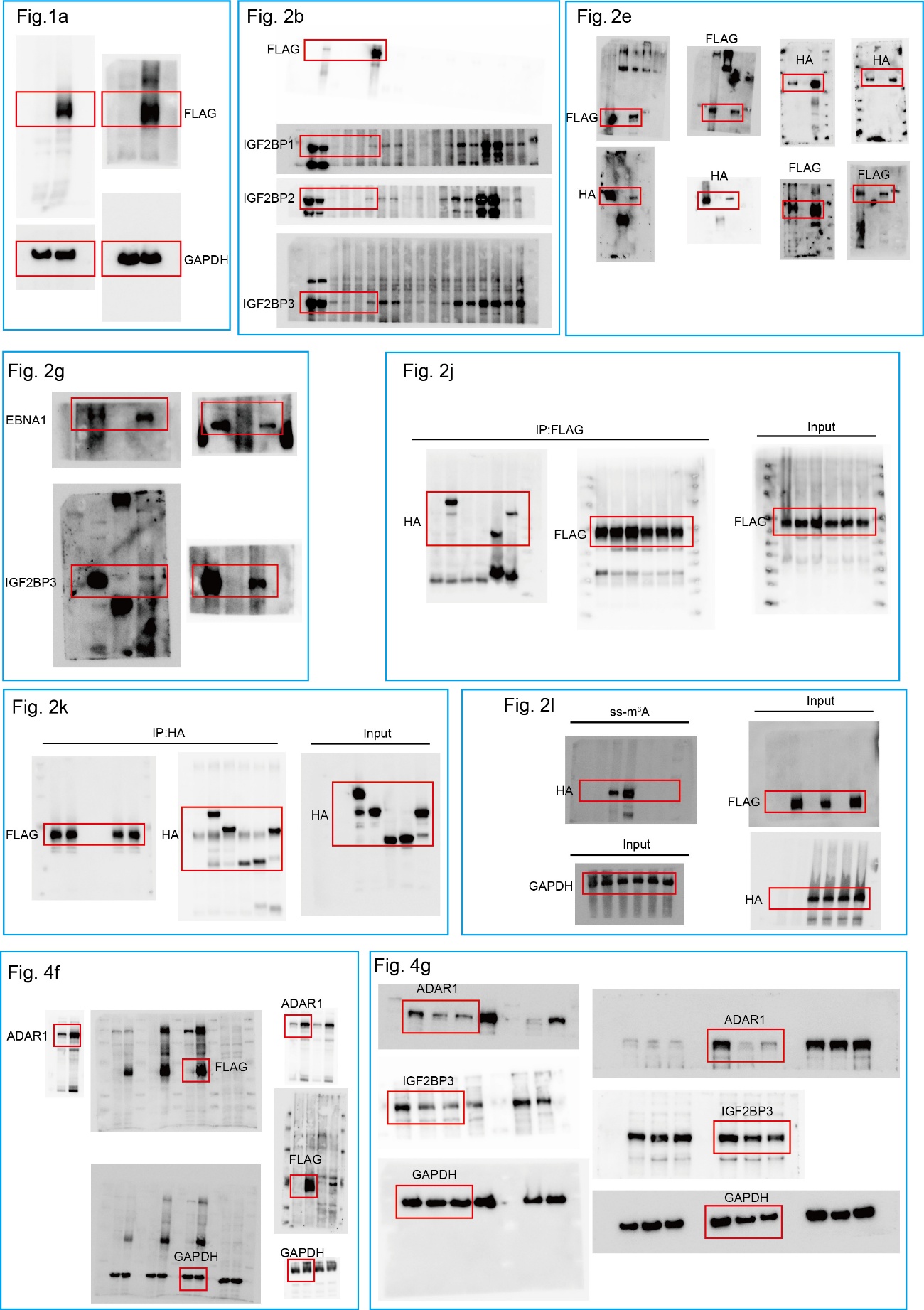


**Supplementary Figure 6. Uncropped western blots for Fig. 1-4.**


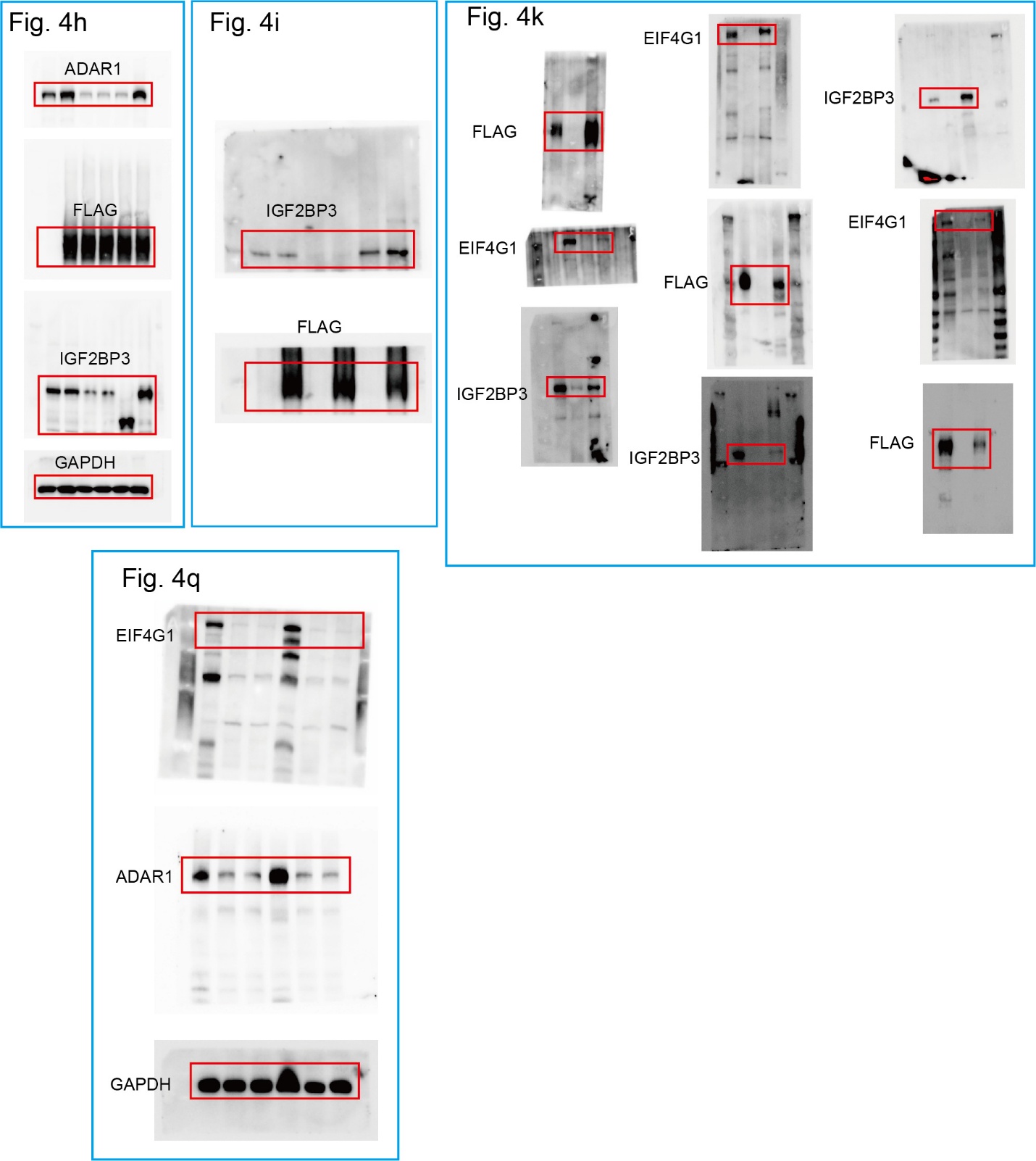


**Supplementary Figure 7. Uncropped western blots for Fig. 4.**


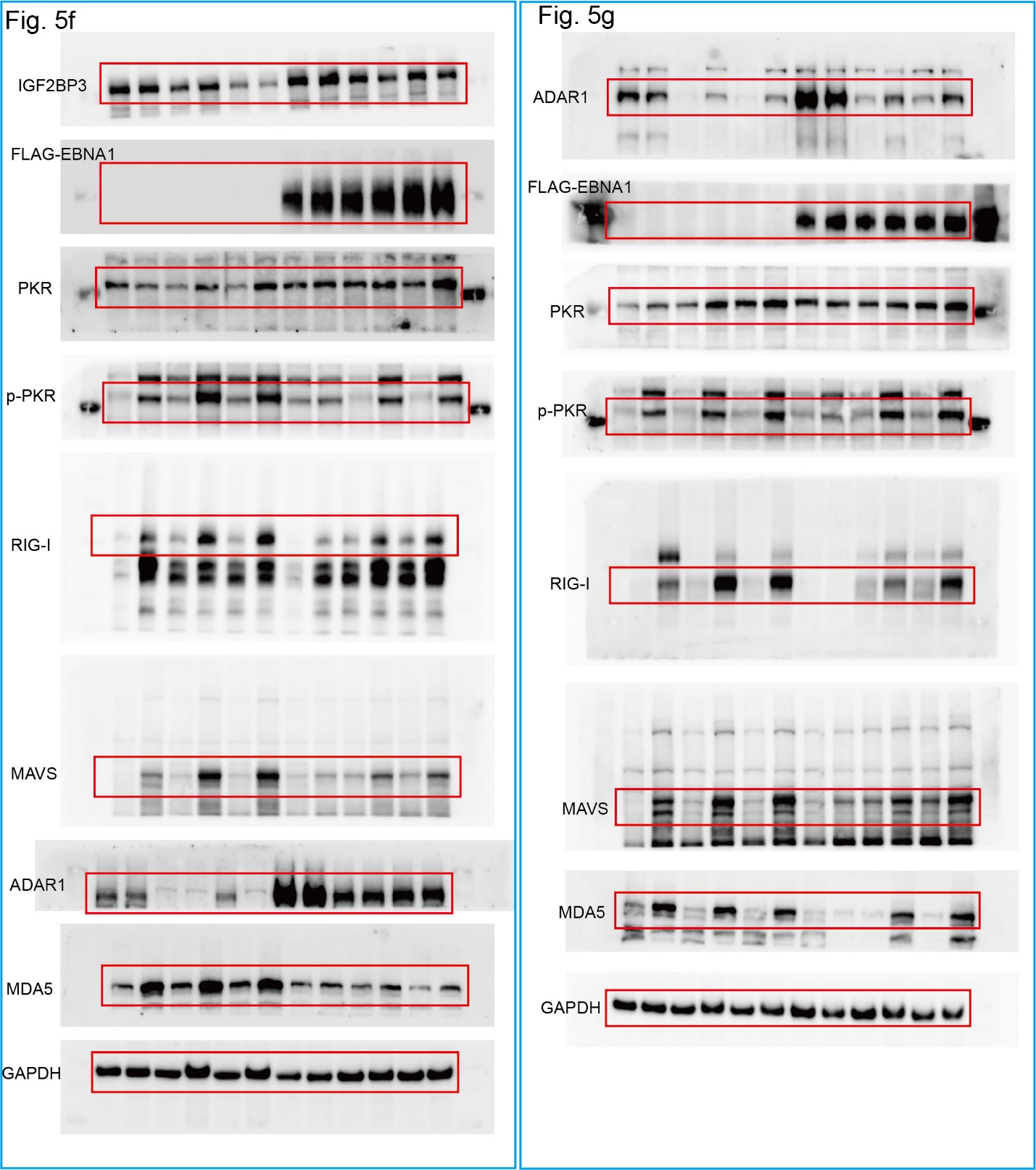


**Supplementary Figure 8. Uncropped western blots for Fig. 5.**


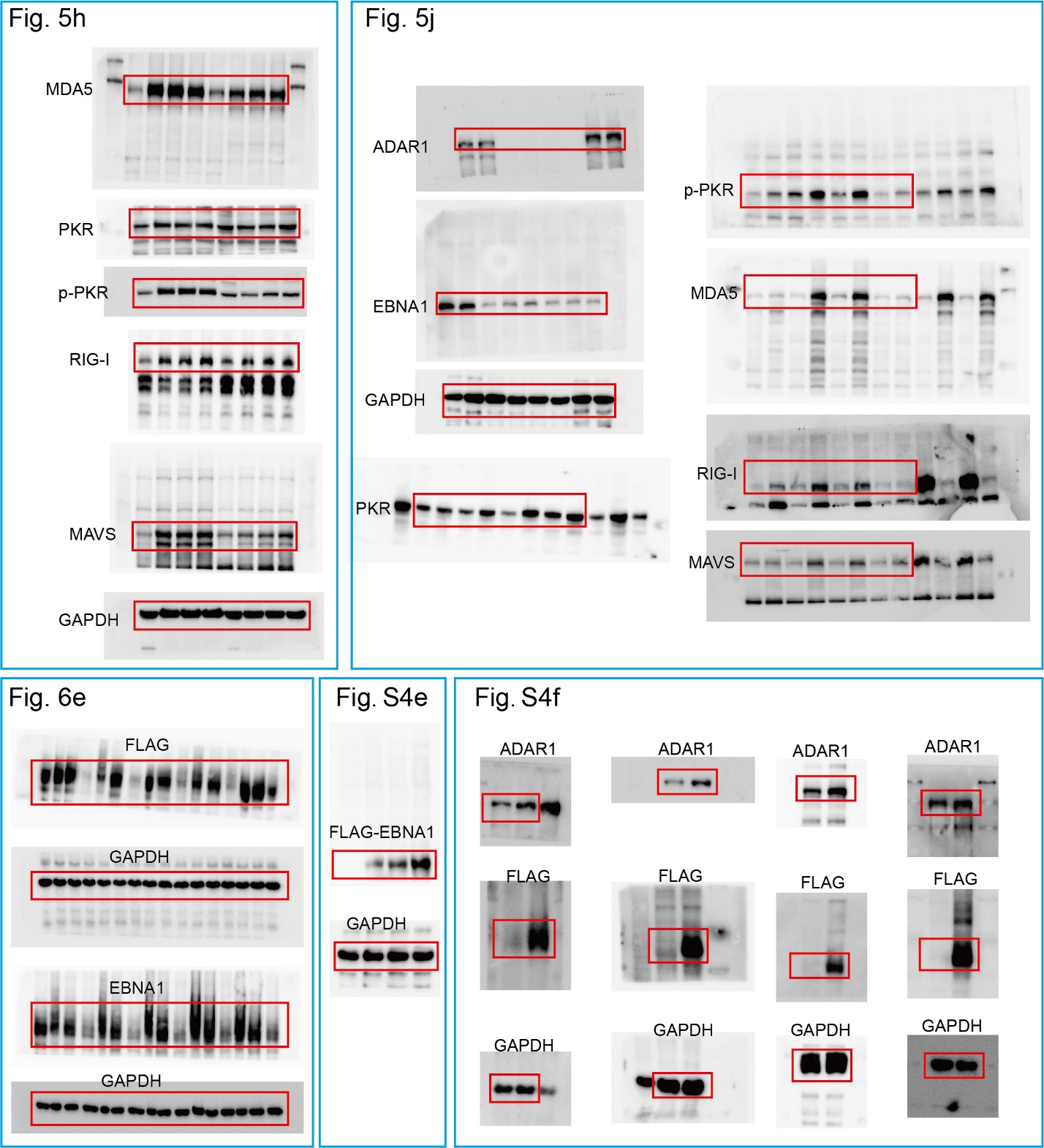


**Supplementary Figure 9. Uncropped western blots for Fig. 5-6, Supplementary Figure 4.**


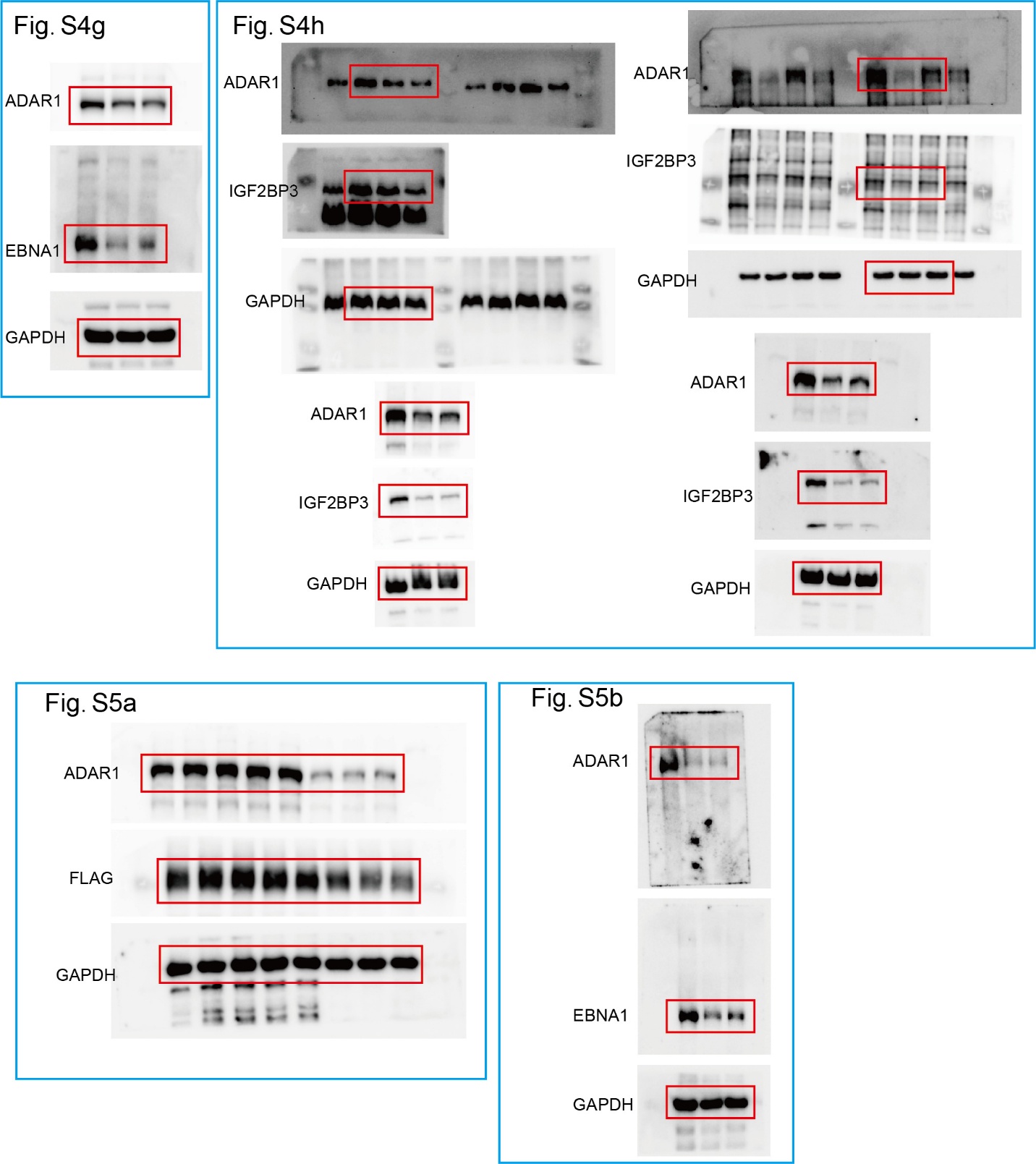


**Supplementary Figure 10. Uncropped western blots for Supplementary Figure 4 and 5.**

Supplementary Table 2.

| **Antigen** | **Fluorophore** | **Clone** | **Company** |
| --- | --- | --- | --- |
| CD45 | FITC | 30-F11 | Biolegend |
| CD3 | Alexa Fluor® 700 | 500A2 | Biolegend |
| CD-11b | Alexa Fluor® 700 | M1/70 | Biolegend |
| CD4 | BV785 | RM4-5 | Biolegend |
| CD8 | BV605 | 53-6.7 | Biolegend |
| CD206 | BV421 | C068C2 | Biolegend |
| F4/80 | BV785 | BM8 | Biolegend |
| CD45 | V450 | 30-F11 | BD Biosciences |
| CD86 | PE | A17199A | Biolegend |
| IFN-γ | PE | XMG1.2 | Biolegend |
