# Supplementary material for: Exogenous Epstein–Barr virus nuclear antigen 1 induces ADAR1-driven tumor resistance against immunotherapy
Source: Signal Transduct Target Ther. 2026 Feb 18;11:63. doi: 10.1038/s41392-026-02574-y (PMC12913968; doi:10.1038/s41392-026-02574-y)
Supplement: Supplementary file 3 — Uncropped Western blots [file 41392_2026_2574_MOESM3_ESM.docx]

Uncropped western blots


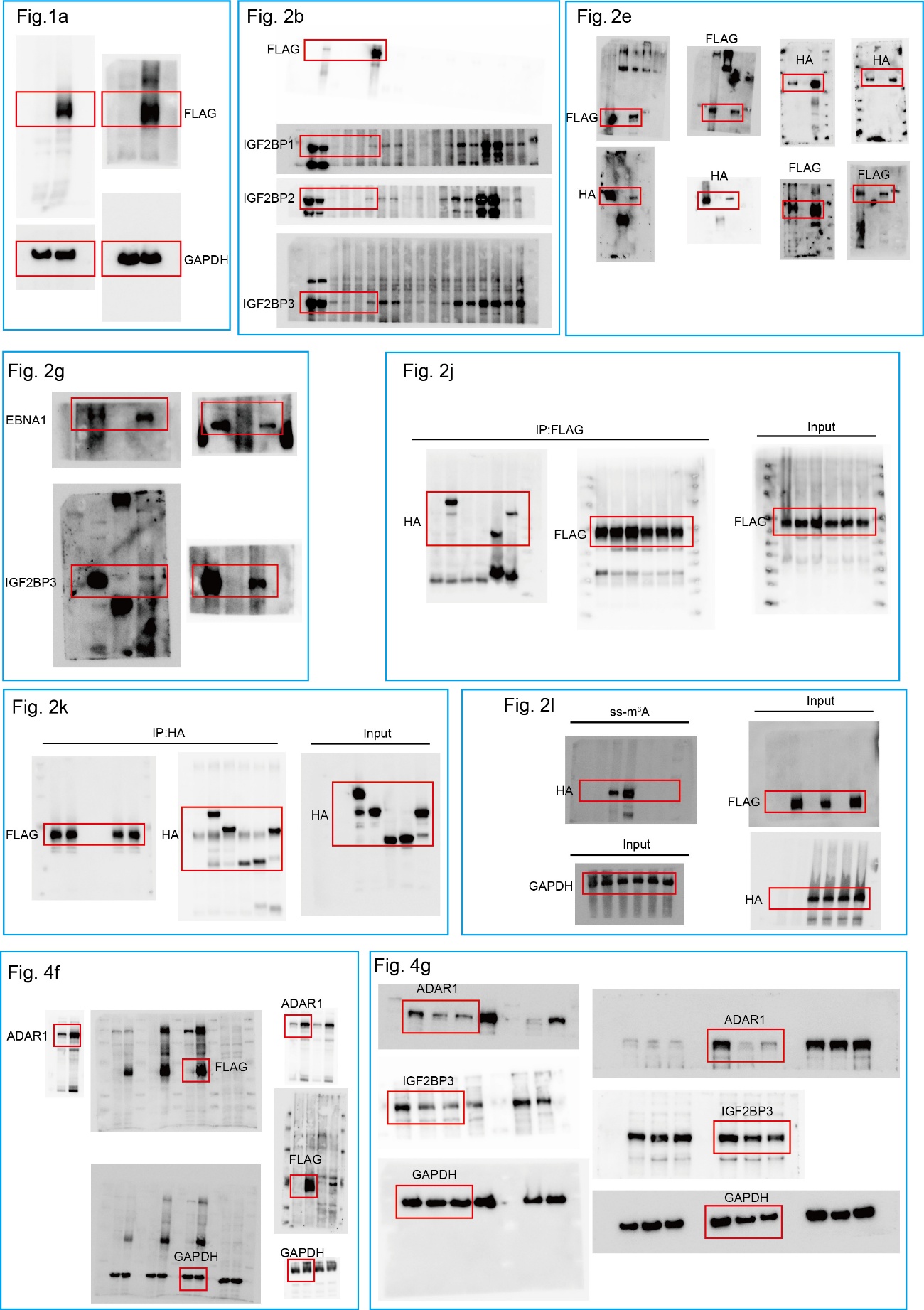


**Supplementary Fig. 6. Uncropped western blots for Fig. 1-4.**


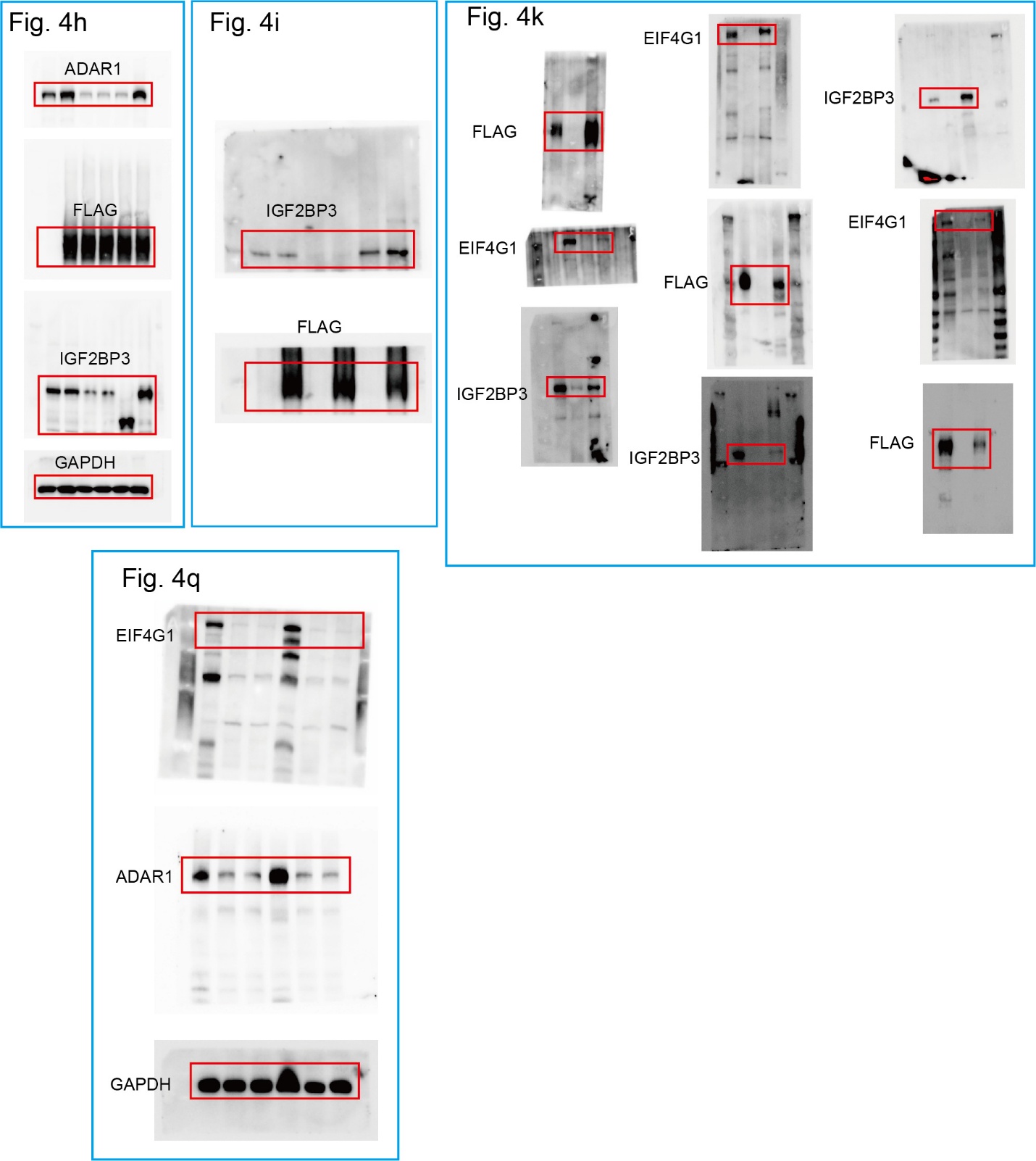


**Supplementary Fig. 7. Uncropped western blots for Fig. 4.**


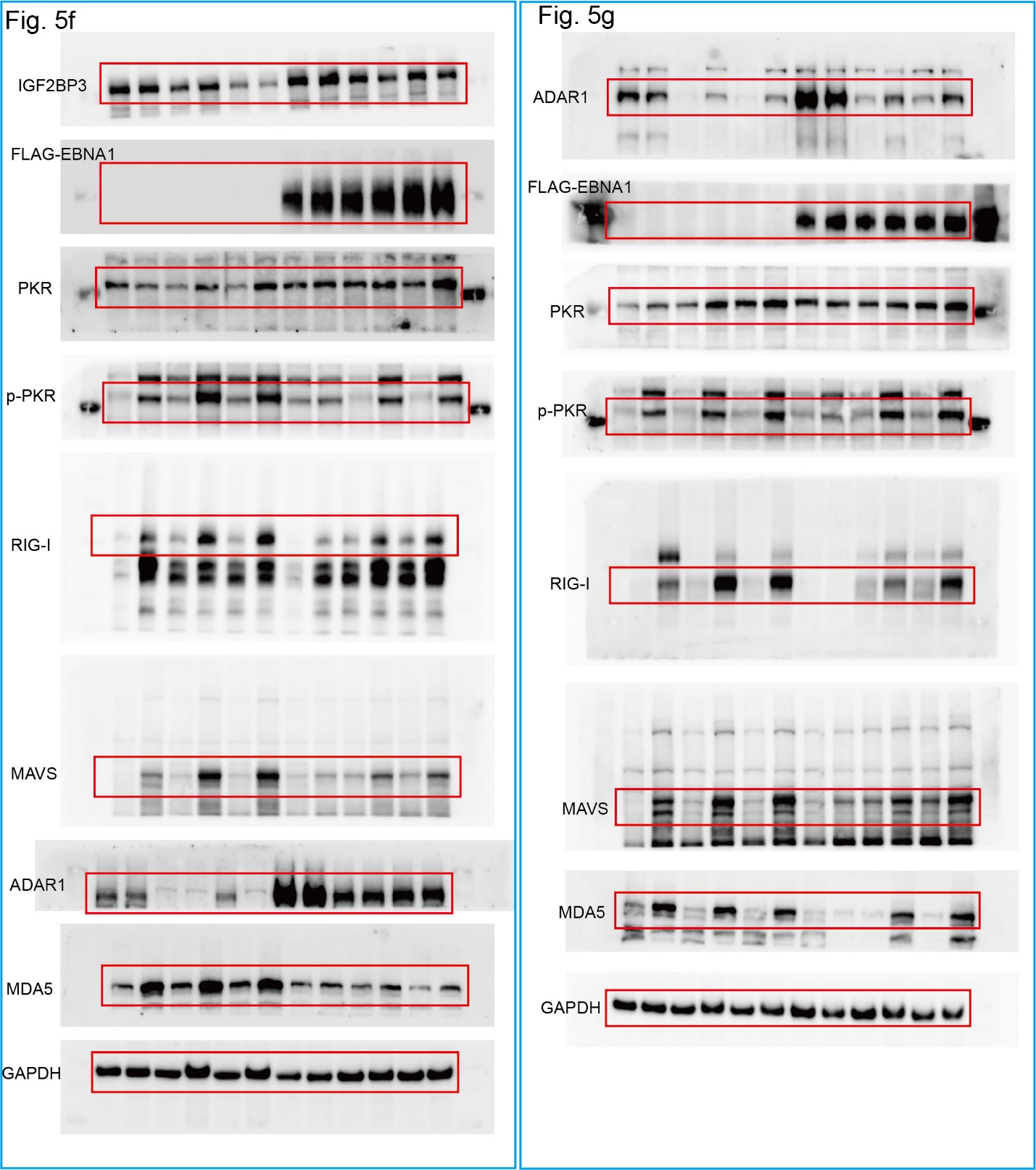


**Supplementary Fig. 8. Uncropped western blots for Fig. 5.**


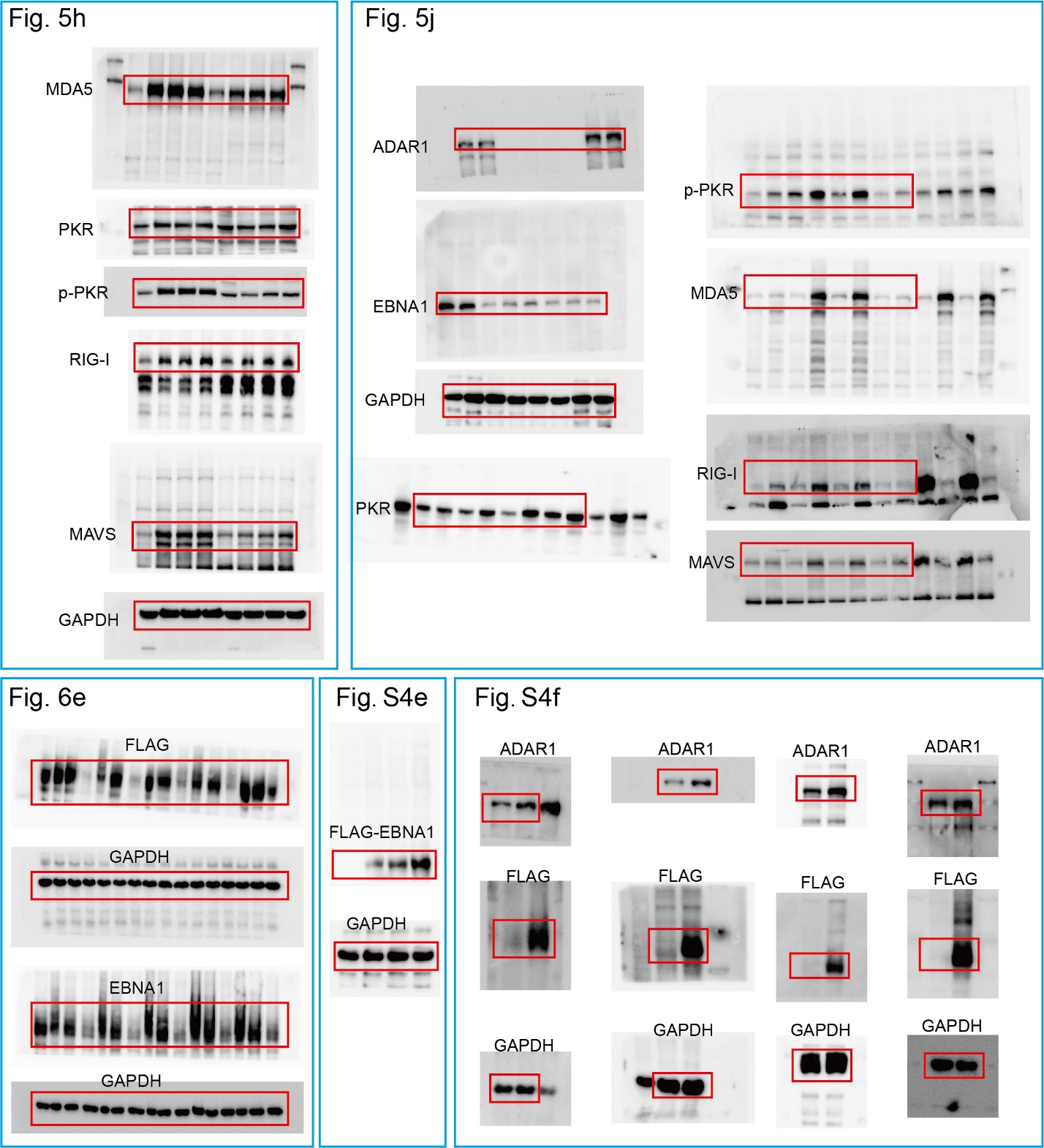


**Supplementary Fig. 9. Uncropped western blots for Fig. 5-6, Supplementary Figure 4.**


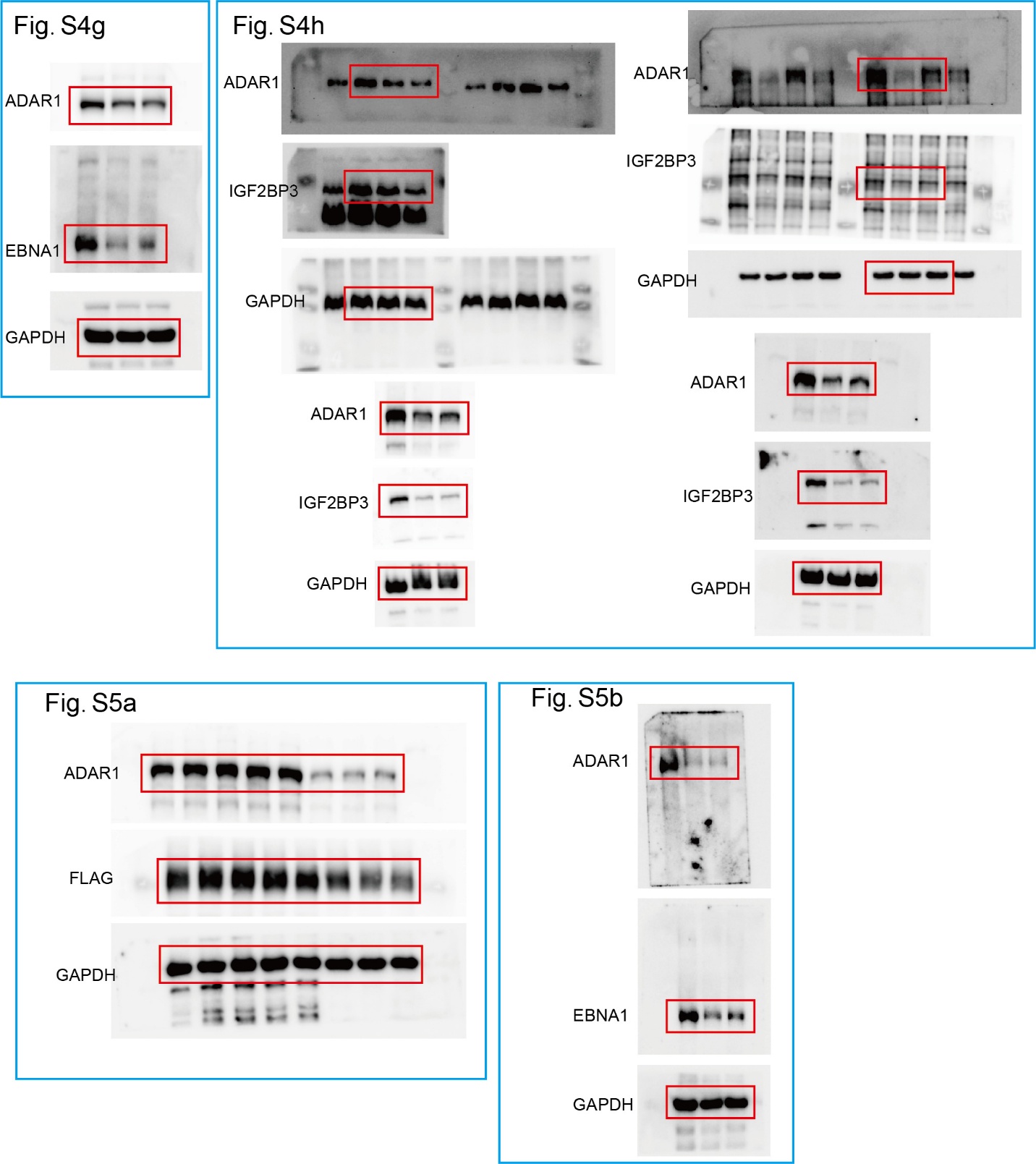


**Supplementary Fig. 10. Uncropped western blots for Supplementary Figure 4 and 5.**
